# STAT3D as a new image-tailored Xenium-based spatial transcriptomics tool for accurately dissecting the tumor microenvironment

**DOI:** 10.1093/nar/gkag937

**Published:** 2026-10-08

**Authors:** Kristi Ajazi, Massimiliano Volpe, Johannes Roylands, Jason Krogh-Pedersen, Kristian Pietras, Lennart Greiff, Malin Lindstedt

**Affiliations:** Department of Immunotechnology, Lund University, Lund 22100, Sweden; SciLifeLab, Department of Immunotechnology, Lund University, Lund 22100,Sweden; Lund University Cancer Centre, Lund 22100,Sweden; National Bioinformatics Infrastructure Sweden, Science for Life Laboratory, Lund University, Lund 22100, Sweden; Department of Immunotechnology, Lund University, Lund 22100, Sweden; SciLifeLab, Department of Immunotechnology, Lund University, Lund 22100,Sweden; Lund University Cancer Centre, Lund 22100,Sweden; Department of Immunotechnology, Lund University, Lund 22100, Sweden; SciLifeLab, Department of Immunotechnology, Lund University, Lund 22100,Sweden; Lund University Cancer Centre, Lund 22100,Sweden; Division of Translational Cancer Research, Department of Laboratory Medicine, Lund University Cancer Centre, Lund 22100, Sweden; SciLifeLab, Department of Laboratory Medicine, Lund University, Lund 22100,Sweden; Department of ORL, Head & Neck Surgery, Skåne University Hospital, Lund 22185, Sweden; Department of Clinical Sciences, Lund University, Lund 22100, Sweden; Department of Immunotechnology, Lund University, Lund 22100, Sweden; SciLifeLab, Department of Immunotechnology, Lund University, Lund 22100,Sweden; Lund University Cancer Centre, Lund 22100,Sweden

## Abstract

Spatial transcriptomics enables researchers to study gene expression within tumor microenvironments at single-cell resolution while preserving tissue architecture. However, analyzing spatial omics data is challenging due to the complexity of the outputs, including large-scale images, millions of transcripts, and numerous spatial coordinates. Moreover, standard volumetric segmentation is often hampered by optical blurring and signal bleed-through in out-of-focus z-planes, which built-in tools in current platforms (e.g. Xenium Explorer) cannot solve. To overcome these challenges, we introduce STAT3D, a transparent, time-efficient, and customizable pipeline for analyzing whole-tumor biopsy slides in a user-tailored manner, including a 3D cell segmentation approach focused on the images’ sharpest z-layers. STAT3D addresses current limitations, such as generic image analysis and uniform downstream processing, by introducing an “image-tailored” strategy that selectively combines only the sharpest optical z-planes to calculate single-cell features and spatial distances. By integrating standard community tools (e.g. Seurat), the pipeline facilitates accurate multiplane cellular segmentation, tailored cell-to-transcript mapping, and fully automated singleR annotation. This versatile tool automatically streamlines critical steps in spatial omics analysis, which facilitates exploration of infiltrating cell types, interactions and tissue architectures by the scientific community.

## Graphical Abstract

**Graphical Abstract:**
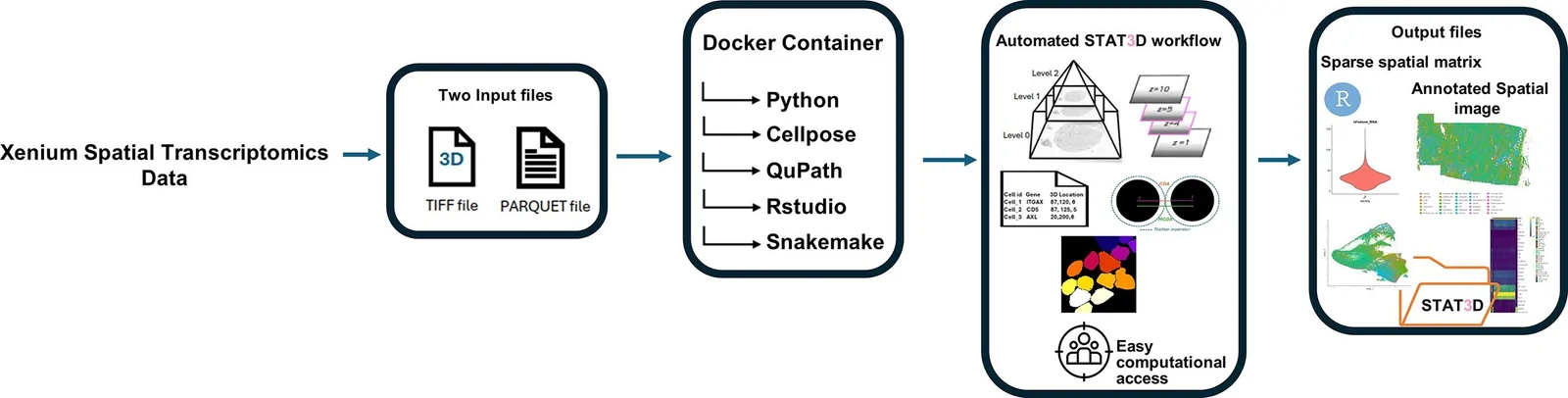

## Introduction

Imaging-based spatial omics technologies reveal critical relationships between genes, proteins, cells, and their spatial organization [1]. Examples include Xenium (10x Genomics), Phenocycler (Akoya Bioscience), and MERFISH (Vizgen) [2]. These technologies bridge the gap between single-cell technologies (e.g. single-cell RNA sequencing, scRNA-seq) and sequencing-based spatial techniques (e.g. NanoString GeoMx and 10x Visium). On the one hand, they are crucial for investigating the tumor microenvironment (TME) and for providing novel approaches to addressing unresolved biological questions. On the other hand, they require significant resources, as spatial omics data comprise large microscopy images (>1 GB), extensive count matrices with millions of transcripts, and diverse metadata [3]. Such requirements increase computational time and require high-performance hardware with extensive memory for downstream analyses, including segmentation and cell-to-transcript mapping. This scenario has led to the development of multiple bioinformatic tools to speed up processing times [4], enhance segmentation algorithms [5, 6], and automate spatial analysis in a single pipeline [7, 8].

Challenges remain, including large image size, customizing parameters, achieving 3D spatial resolution, and ensuring unified container applications. Allowing users to specify features such as nuclei diameters and nuclear expansion (NE) before downstream analysis steps (e.g. cell annotation) can drastically improve accuracy and ensure precise transcript-to-cell associations. Achieving accurate spatial resolution with 3D cellular segmentation is crucial for understanding the TME architecture and for distinguishing rare cell subpopulations in cell-dense biopsies [9]. The method described in this paper performs unconventional, nonfull-volumetric, 3D cellular segmentation. Rather than segmenting the entire volume, we extract only the sharpest z-layers from the spatial transcriptomics image to construct a new image, which we then 3D segment. The choice of more crisp z-layers is guided either by Laplacian score (LS) measurement [10] or, alternatively, by direct user input.

Examples of rare immune cells of particular interest include conventional dendritic cell type 1 (cDC1s) and LAMP3+ dendritic cells (also known as mature regulatory DCs), which are often described as antigen-experienced DCs with regulatory traits that may support antitumor immunity depending on the TME and cancer type [11–15]. Among the rare immune cell types in the TME, mast cells and B cells have also been reported to play multifaceted roles during tumor progression [16–20]. Understanding their spatial organization may illuminate unexplored mechanisms driving tumor immune escape and treatment failures. Prior studies, using spatial proteomics and spatial transcriptomics, have attempted detailed 3D analyzes of tumors’ cellular architecture and interactions [7, 21, 22]. However, restrictions such as limited antibody panel coverage, constrained imaging areas, and complex image formats hinder the detailed spatial resolution of rare myeloid cells within the TME.

From a computational perspective, it is essential to provide containerized applications (e.g. via Docker) [23] to implement automated steps, ensure reproducibility, and reduce variability across tools and operating systems (e.g. Mac, Windows, Linux). It enables a broader range of scientists, not just bioinformatics experts, to easily access and perform complex computational analyses. In this paper, we introduce STAT3D (Spatial Transcriptomics Analysis Tool in 3D), a pipeline designed to accelerate computational processing, reduce image sizes, customize image analysis, support maximum intensity projection (MIP), and perform 3D or 2D cell segmentation using Cellpose (version 2.1) [17]. Additionally, it facilitates cell-to-transcript mapping, all encapsulated within a single Docker container. STAT3D operates on pyramidal OME-TIFF images from both imaging and sequencing-based spatial omics platforms. Due to the multiple resolution levels, the size of OME-TIFF images can be substantial, with each level containing a defined number of z-stacks, resulting in large images and increased processing times. Notably, the resolution of OME-TIFF images is inversely proportional to the number of levels, thus, the lower the level, the higher the resolution (Fig. 1A). We tested STAT3D using four spatial transcriptomics datasets generated with Xenium platform. Moreover, we demonstrated the role of STAT3D in the resolution of immune cell clusters and in gene expression profiling. We eventually validated STAT3D’s predictions of rare myeloid cluster localizations using multiplex immunofluorescence (mIF) assay. The bioinformatics packages, programs, and libraries required for STAT3D’s functionality are hosted in a Docker container available in the STAT3D repository (https://github.com/kristiajazi/STAT3D). This computational format enables users with varying degrees of bioinformatic expertise to launch the pipeline easily with a single command line via the Snakemake workflow management system [24]. STAT3D marks a significant advancement towards faster, customizable, and automated spatial transcriptomics analysis, effectively overcoming the “one-size-fits-all” standard workflows and represents a valuable resource for the spatial omics community.

**Figure 1.**
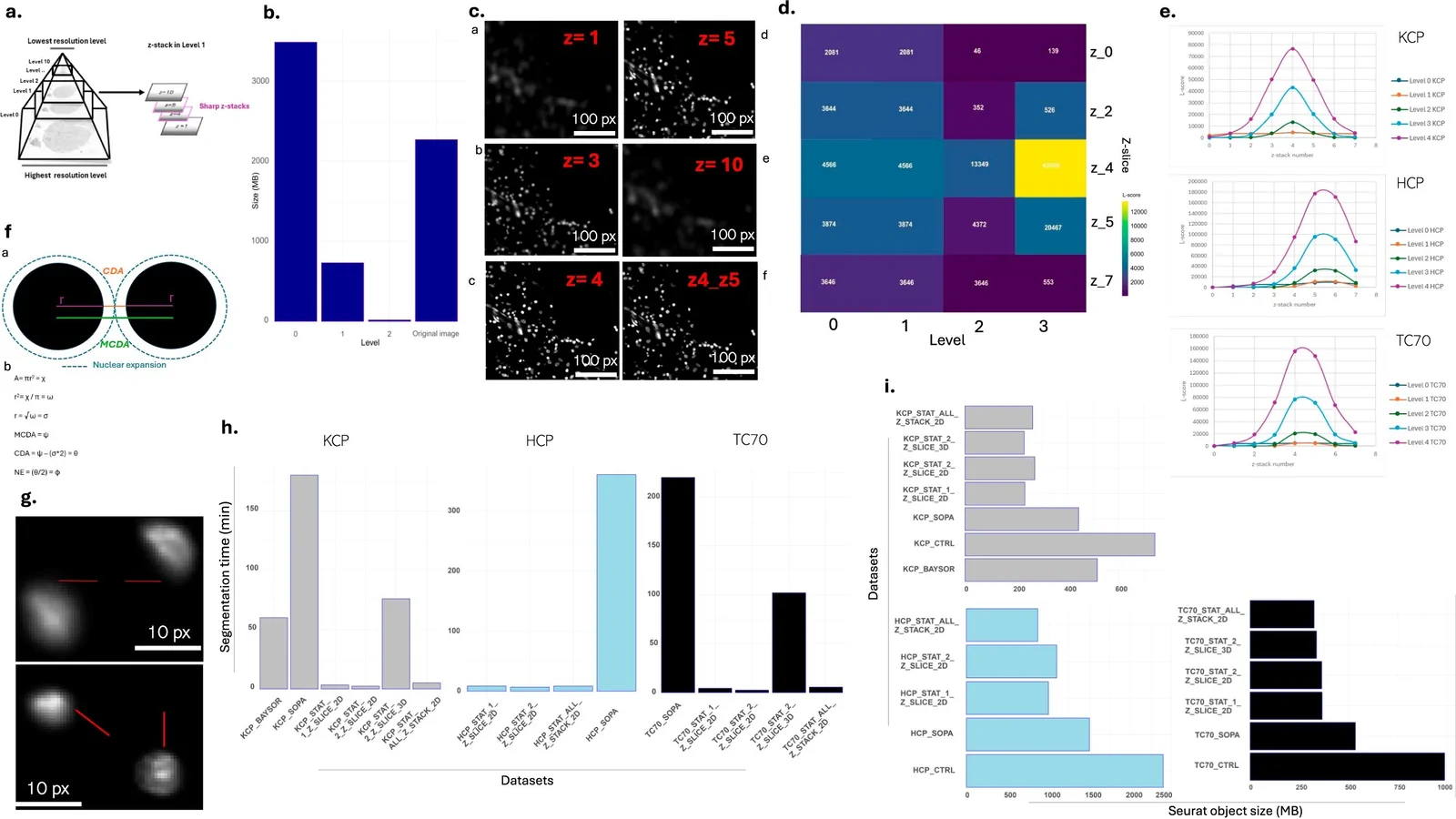
STAT3D image analysis in KCP, HCP and TC70 datasets. (**A**) Schematic representation of one pyramidal OME-TIFF image and the structure of its levels and z-stacks. (**B**) Bar plots displaying size differences (*y*-axis) for three different levels (0, 1, 2) and the original image (*x*-axis) in the KCP dataset. (**C**) QuPath images of sharp (c, e) and blurry (a, b, e) z-slices within the z-stack of level 2 in the KCP dataset. QuPath image representation of the merged sharp z-stack image (f). (**D**) Heat map displaying LS for five z-slices (z0, z2, z4, z5, z7 on the *y*-axis) across four different image levels (*x*-axis) in the KCP dataset. (**E**) Line plots displaying L-scores of the first eight z-slices across the first eight levels of the pyramidal image in the three datasets (KCP, HCP, TC70). (**F**) Schematic representation of STAT3D method for nuclei diameter and NE measurement (a). Example of mathematical calculations for nuclei diameter and NE measurements (b). (**G**) QuPath image from the KCP dataset displaying NE distances at 12 pixels (red lines). (**H**) Segmentation runtime performance across KCP (gray), HCP (light blue), and TC70 (black) datasets. Bar plots show total segmentation time (minutes) required for each dataset under identical computational conditions (GPU used for all settings except Baysor, which used CPU). (**I**) Bar plots illustrating memory storage demands in KCP (gray), HCP (light blue), and TC70 (black) datasets. Bar length represents size (MB) of each Seurat object.

## Materials and methods

### Device specification

The STAT3D pipeline was developed and run on a 13th Gen Intel(R) Core (TM) i7-1355U (1.70 GHz) processor with 32 GB of installed RAM, using a dedicated Graphics Processing Unit (GPU) (NVIDIA RTX A500, version 573.44) and a 64-bit operating system (Windows 11). STAT3D was tested on Alvis high-performance cluster at Chalmers Centre for Computational Science and Engineering (C3SE).

### Datasets used

The validity of the STAT3D pipeline was assessed using four Xenium datasets derived from whole human formalin-fixed paraffin-embedded (FFPE) biopsies. These samples were analyzed using a human multitissue panel comprising 50 custom-designed probes targeting 477 genes in total. The three publicly available datasets used were the “Pancreatic Cancer with Xenium Human Multi-Tissue and Cancer Panel” labeled as “HCP”, the “Kidney Cancer (papillary renal cell carcinoma, PRCC) Xenium Human Multi-Tissue and Cancer Panel”, labeled as “KCP” and the “Human non-diseased Kidney (Xenium Human Multi-Tissue and Cancer Panel)” labeled as “HD”. Additionally, we included our in-house human tonsillar cancer (TC) dataset, termed “TC70”.

### Pipeline implementation and reproducibility

Reproducible execution was ensured through deterministic environment resolution using the *pixi.lock* file in combination with the *–frozen* flag during Docker builds. STAT3D pipeline performed runtime validation of required configuration parameters and automatically generated a Quarto [25] HTML report to support transparent and reproducible benchmarking.

We generated the Snakemake workflow DAG (Directed Acyclic Graph) and rendered it as a PNG using Graphviz in a single command, omitting the first line of configuration metadata to ensure correct parsing (Supplementary Code).

### Generation of a smaller 2D or 3D image

HCP, KCP, and TC70 input images were large pyramidal Open Microscopy Environment Tagged Image File Format (OME-TIFF) files of 3.32 GB, 2.21 GB, and 2.2 GB, respectively, containing up to 13 levels of resolution and up to 13 z-stacks per level (Table 1 and Fig. 1A). Levels before and above the third produced blurry images (Supplementary Figs S1–S3), where only two images (∼16.5%) in the z-stack remained sharp. Therefore, STAT3D first extracted one of the highest resolution levels (e.g. level 2), which was computationally lighter than either the original image (comprising all levels) or level 0 (Fig. 1B), creating in parallel, a new folder containing all the images of the z-stacks. Secondly, within the extracted level, STAT3D enabled the selection of different combinations of z-slices (e.g. from one z-slice to six z-slices) or the whole z-stack selection. STAT3D pipeline automatically merged the two sharpest z-slices of the z-stack, “z_top1” and “z_top2” (e.g. z 4 and z 5, Fig. 1C), discarding the rest (Fig. 1C resulting in a new smaller two-planes OME-TIFF image (Table 2) suitable for 3D Cellpose nuclei segmentation. The choice of single z-slice resulted in a single z-plane image that fitted the 2D segmentation format. STAT3D offered whole-z-stack image resizing for large datasets using the MIP method via the NumPy library. With MIP the entire 3D z-stack image was converted into a 2D image considering the brightest pixel (voxel) in each z-slice and displayed that pixel intensity value in one final 2D smaller OME-TIFF image [26, 27]. Ultimately, STAT3D produced a lighter NumPy array representing a 2D (single-stack) or 3D (multistack) image, with gray intensities, 16-bit unsigned integers, and a tile size of 1024 × 1024, utilizing JPEG2000 compression and ‘YCX’ or ‘ZYCX’ data dimensions’ order (Z = slices, Y = rows, C = channels, X = columns). In this process, the images’ metadata were read and stored as 2D or 3D TIFF files using Python libraries TIFFile [28] and NumPy [29] and saved in OME-TIFF format.

**Table 1.** Structure of OME-TIFF pyramidal images in HCP, KPC, and TC70 datasets

Dataset	Total number of levels	Total number of z-stacks in each level
HCP	13	13
KPC	11	11
TC70	11	11

**Table 2.** OME.TIFF image size improvement with STAT3D

Dataset	Original image (MB)	Level 2 image (MB)	Level 2 z_top1 and z_top2 (MB)	Level 2 z_top1 (z = 4) (MB)	Level 2 All_z (MIP)
HCP	3399.68	308	44	-	23.14
KCP	2263.04	188	24.81	11.71	-
TC70	2293.76	179	26.29	-	-

Z-stack sharpness was quantified by adopting the LS (or L-score, Fig. 1D) [30]. The LS mathematically defined the changes in pixel intensities between the original image, level zero, level 1 and level 2 at z1, z3, z4, z5, and z10 stacks. Higher LS was associated with higher image sharpness. STAT3D’s run included the display of the L-scores and created an additional CSV file for the automatic or user-chosen z-stacks combination.

### Custom-based image analysis

QuPath (V.0.5.1) and R (RStudio 2024.12.0 + 467) were used in the STAT3D pipeline to calculate cell diameter and NE (Fig. 1F). This step involved analyzing full-image annotations using the QuPath “Cell Detection” function, with a 10-pixel background radius, 1-pixel sigma, 2-pixel cell expansion, and an intensity threshold of 30. Measurements of each nuclei area and centroid coordinates (x and y) were exported in Excel Comma Separated Values File (.csv) format for analysis in R. This step requested the installation of the following R libraries: *dyplr, readr, yaml*, and *geosphere*, which enabled data manipulation and cleaning, data reading, YAML (“Yet Another Markup Language”) files reading into R objects, and spatial distance calculation between two points, respectively. The centroids indicated the centers of the nuclei. First, the pixel values were converted into µm measurements based on the image resolution level. Next, the average area for all nuclei was calculated using the formula A = πr^2^, where “A” is the average area of the nuclei. STAT3D computed the nuclear diameter average (NDA) to obtain r. Subsequently, STAT3D determined the centroids’ minimum distance via *dist* R function, with the Euclidean approach. The resulting R object of class “dist” was converted into a full matrix via the *as.matrix* function. To exclude the zero distance to itself, STAT3D set the diagonal of the new matrix as infinite (*Inf*) before calculating the minimum distance across all centroids with *apply* function. Thereafter, STAT3D calculated the average minimum distance between all centroids (MCDA). STAT3D obtained the nuclei distance average (NDA) by subtraction of doubled r values from the MCDA number. To conclude, the NE was half of the CDE value (Fig. 1f). The NDA and NE parameters represented, respectively, the customized values required for subsequent steps in of the STAT3D pipeline, such as 3D or 2D segmentation and cell-to-transcripts mapping.

### Nonconventional 3D and 2D cellpose spatial segmentation

STAT3D achieved 3D and 2D Cellpose-based cellular segmentation using a pre-trained *nuclei* model, based on the image-tailored NDA measurement identified with QuPath. The segmentation was performed on the new, lighter image file “morphology.ome._STAT3D.tiff” generated in the first step of the pipeline, and by setting the Cellpose Boolean parameter *do_3D* as “true” or “false”. STAT3D enabled truly image-tailored cell segmentation via the parameter “nuceli_diameter” which allowed for user-specified values including decimal numbers. The segmentation process was accelerated by enabling the processor to take full advantage of the GPU through the installation of the PyTorch framework [31], which allowed STAT3D to combine the GPU-accelerated libraries from Torch and PyTorch deep neural network models. Users without a dedicated graphics card can still run STAT3D in CPU mode (“cellpose_use_gpu: false”). As a result, STAT3D generated a NumPy dictionary file (“.ome_seg.npy”) and a series of TIFF images representing the segmented masks (“.ome_cp_masks.tif”), where each mask corresponded to cell nuclei.

### Cell-to-transcripts mapping

The STAT3D workflow incorporated Python libraries such as Pandas [32], NumPy, Re [32], and SciPy [33] in the cell-to-transcript step. Here, the transcripts file (“transcripts.parquet”) and the segmented image (“.seg.npy”) were converted into a gene expression matrix for each segmented cell, rebuilding the classic cell-gene count matrix. First, STAT3D loaded the cells’ segmentation mask (“mask array”), which was a 3D NumPy array in z, y, x coordinates, where each pixel was labeled with an integer. Here, many pixels labeled with the same integer represented a unique cell. Secondly, STAT3D proceeded by loading transcript data from “transcript.parquet” file and creating a unique features list and lookup dictionary where an index was assigned to each unique feature. Thirdly, STAT3D initialized a Gene x Cell count matrix, extracting cell identities (IDs) from the mask array file. This matrix, initially loaded with zeros, was then filled with transcripts counts. Lastly, the cell-to-transcripts mapping matched each transcript’s coordinates with the appropriate cell ID based on the pixel position in the mask array (e.g. cell_id = mask_array[round(z_slice)][round(y_pixel)][round(x_pixel)]). STAT3D ensured that unassigned transcripts located far from any nuclei were not lost or associated with the wrong cell IDs by applying nuclei expansion search. This step defined a 2D or 3D area around the displaced transcript within which the algorithm searched for nearby cells. The region around the transcript was defined based on the previously generated NE value from STAT3D in QuPath. This produced a sparse 2D or 3D spatial count matrix, with feature names as rows and cell IDs as columns. To collect spatial information and generate a features-barcodes matrix, a list of coordinates was introduced to summarize the 2D or 3D location of each cell and its associated transcript. As a result, the 2D or 3D cell-to-transcripts mapping step produced one barcode file containing cells’ 2D or 3D spatial coordinates, one feature file listing all gene transcripts and one sparse gene expression matrix. These files formed the basis for generating the Seurat spatial transcriptomics object in the final step of the STAT3D pipeline.

### Seurat spatial transcriptomics object

STAT3D took advantage of R programming language and its integrative multimodal packages such as *Seurat v5* to create one Seurat spatial object. STAT3D used “Read10x” function to read in the GNU Zip (.gz) outputs generated by the cell-to-transcripts mapping step and return a unique molecular identifier count matrix. The row and column values of the count matrix were, respectively, the numbers of molecules per feature and the cell IDs. Next, STAT3D created a Seurat object with “CreateSeuratObject” function from *Seurat v5* package and its core data structure package *SeuratObject*. This object aims to save both gene counts and subsequent single-cell information, such as dimensionality-reduction data. STAT3D implemented the Seurat object, adding spatial coordinates and cell IDs. This information was retrieved from the Tab-Separated-Values barcodes file (barcodes.tsv) produced automatically by the pipeline at the cell-to-transcript mapping step. The cell IDs and spatial coordinates (in cell_id, X, Y order) were renamed as “spatial_0”, “spatial_1”, and “spatial_2″. These labels were necessary for the development of the spatial object for dimensional reduction (“DimReduc”). To align with the DimReduc structure requirements, STAT3D used the Matrix package to convert the two columns of the data frame “spatial_1” and “spatial_2” into a plain numeric matrix, where each row was labeled with cell IDs. Eventually, STAT3D created the spatial dimension-reduced object after computing “CreateDimReducObject” function. Here, the embeddings were characterized by cell IDs as rows, spatial coordinates as columns, and “spatialobj_” as key prefix for the plots or dimensions’ labels. STAT3D assigned the spatial object to the initial Seurat object under a new slot called “spatialobj_” and located in the “reductions” column. In conclusion, STAT3D generated a Seurat spatial object stored in R data serialization format (.rds), along with a portable document format (.pdf) to enable the visualization of the tissue’s first spatially reduced transcriptomics image.

### Spatial transcriptomics data analysis and automated annotation

The data were processed using R version 4.4.3, Seurat version 5.1.0. The spatial object was generated with the “CreateDimRedObject*”* function. Expression data were normalized using SCTransform [34], principal component (PC) analysis considering the first 30 PCs [35], UMAP dimensionality reduction [36], cells clustering with Shared Nearest Neighbor graph [37] using the first 30 PCs and applying the Louvian algorithm [38]. The identification of differentially expressed genes for each of the identity classes in the datasets was achieved with the Seurat function “FindAllMarkers”. STAT3D supported automated annotation through the R package *singleR* [39]. The implementation of *singleR* was achieved by installing supplementary R packages, as *celldex* [39] and *scRNAseq* [40]. STAT3D’s SingleR-based annotation provided curated cell type labels and reference expressions from six reference datasets such as “Human Primary Cell Atlas Data”, “BlueprintEncodeData”, “DatabaseImmuneCellExpressionData”, “MonacoImmuneData”, ”MouseRNAseqData”, and ”NovershternHematopoieticData”. STAT3D allowed for SingleR “main” or “fine” cell types labeling via a customized parameter. Ultimately, STAT3D returned one UMAP plot displaying all the SingleR-annotated clusters, one spatial UMAP graph projecting the labeled clusters onto the tissue biopsy and a quality control heatmap showing prediction scores for each cell type.

### Fast nearest neighbor analysis

Cell separation efficiency in crowded cell populations was assessed using the get.knn function from the Fast nearest neighbor (*FNN*) package in R [41]. The HCP, KCP, and TC70 datasets were analyzed with “kd_tree” algorithm to achieve accurate nearest neighbor distances between SingleR-annotated T cells and B cells. These distances were used to calculate the percentage of T cells located within a 20 µm radius of each B cell.

### Gene enrichment analysis

Functional profiling of the genes was performed using the g:GOSt (g:Profiler) [42]. Here, the statistical enrichment analysis was based on Fisher’s one-tailed exact test to identify over-represented Gene Ontology (GO) terms. The g:GOSt p-value reflected the likelihood that the overlap between the query and a given ontology term occurred by chance, and the probability of the observed interaction. The GO acyclic graph structure included three major subontologies: “Molecular Functions” (MF), “Biological Process” (BP), and “Cellular Component”. Moreover, g:GOSt analysis incorporated alternative data sources focused on biological pathways (e.g. KEGG, WikiPathways), regulatory DNA motifs (e.g. MIRNA), and protein complexes or annotations (e.g. CORUM, Human Protein Atlas).

### Immunohistochemistry validation

Samples for immunohistochemistry (IHC) validation were obtained from the Department of Otorhinolaryngology, Head & Neck Surgery, Skåne University Hospital (Sweden). The antibodies used were validated using human TC FFPE samples sectioned at 4 µm. The validation was performed with 3,3′-diaminobenzidine staining, using the ImmPRESS horseradish peroxidase PLUS Polymer Kit (catalogue numbers MP-7802, MP-7801, and PI-9400). After deparaffinization and rehydration, the samples underwent heat-induced antigen retrieval in citric acid buffer (pH 6) at 110°C for 30 min using a TintoRetriever pressure cooker (Cancer Diagnostics). Chromogenic IHC staining was performed manually following ImmPRESS^®^ guidelines, using antibodies against Cytokeratin (HistoLab, AE-1/AE-3) at a dilution of 1:300, LAMP3 (Abcam, ab1010E1.01) at 1:50, CD8a (Thermo Fisher Scientific, OTI3h6) at 1:1000, CCR7 (Abcam, ab253187) at 1:250, and IDO1 (Abcam, Ab211017) at 1:500. Staining intensity was evaluated using a PhenoImager HT (1.0.13, Akoya Bioscience^®^, Marlborough, MA) microscope at 20× and 40× magnification.

### Immunofluorescence with PhenoImager HT

mIF was conducted on sequential FFPE slides of TC70 sample. The slides were stained using multiple rounds of tyramide signal amplification with the Opal 7-plex manual detection kit (catalogue number NEL797001KT, Akoya Bioscience). For all markers, the following were included: 4′,6-diamidino-2-phenylindole (DAPI), Opal Polaris 480, Opal Polaris 520, Opal Polaris 570, Opal Polaris 620, and Opal Polaris 690, each utilized in combination with specific antibodies. Table 3 shows the experimental design for the mIF. The PhenoImager HT scanner was used to image the stained slides and capture fluorescence images. Image analysis was performed using QuPath (V.0.5.0).

**Table 3.** mIF experiment layout

Target	Opal	Acquired color
CCR7	690	Red
LAMP3	520	Green
IDO1	480	Turquoise
CD8	570	Yellow
Cytokeratin (PanCK)	620	Orange
Nuclei	DAPI	Blue

### Spatial analysis of mIF assay

The data analysis of mIF images was performed with Seurat (V 4.4.3) and SPIAT software package [43] included in Bioconductor open-source software for bioinformatics (V 3.22).The raw data were stored in one QuPath file in CSV format. The categorization of TC70 immune microenvironment with SPIAT relied on the calculation of spatial data in the form of X and Y coordinates for every single cell, their marker intensities and cells’ phenotypes. To infer interaction between different cell types, the cross-K function (Kˆ) (equation 1), and the normalized mixing score (NMS) (equation 2) were adopted. Kˆ described the architecture of two proximal cell clusters of interest (i and j) within a region of interest (ROI) for a set number of radii (100). The proximity between the two cell clusters was compared to a Poisson distribution, assuming independent distributions of the two immune cell types of interest. Cross-K function values above the Poisson distribution indicated cluster formation, while values below the Poisson distribution indicated clusters’ spatial separation.


(1) \begin{eqnarray*}
{\rm K}{\hat i}j (r)=\frac{A}{n_i\,\,n_j}\mathop {\sum\nolimits_{k=1}^{n_i}}\mathop\sum\nolimits_{l=1}^{n_j}{}^{I(d_{kl}\le r)}/W_{kl}
\end{eqnarray*}

Equation 1. *A*: area of the ROI; *ni and nj*: number of points/cells of the respective category (i/j); *dkl*: Euclidean distance between cells of the respective category (i/j); *I(dkl ≤ r)*: indicator function that is equal to 1 as long as the distance between the two cells is less than or equal to r, otherwise it is equal to 0; *wkl*: correction factor equalizing edge effects stemming from bordering cells.

The normalized mixing score (NMS) function included in the SPIAT package was adopted to quantify the number of interactions between a reference and a target cell cluster within a specified radius. Higher NMS scores reflected greater mixing of the two cell clusters of interest. The normalised score accounted for the number of target and reference cells in the ROI.


(2) \begin{eqnarray*}
NMS = \frac{1}{{{n_i}}}{\mathrm{\,\,}}\mathop \sum \nolimits_{{\mathrm{k}} = {\mathrm{l}}}^{{n_i}} I({d_{kl}} \le r)/{n_j}
\end{eqnarray*}

Equation 2. *n_i_* and *n_j_*: number of points (or cells) of the respective category (*i/j*); *d_kl_*: Euclidean distance between cells of the respective category (*i/j*); *I*(*d_kl_* ≤ *r*): indicator function, that is equal to 1 as long as the distance between the two cells is less than or equal to *r*, otherwise it is equal to 0.

The SPIAT-formatted image object was generated from the mean fluorescence intensities of single cells, their x- and y-coordinates, and cell classifications, contained in the mIF data and extracted from QuPath as a CSV file. First, the dataset was screened for signatures of up to three markers (Supplementary Table S4), followed by annotation of each marker-derived cell population. Second, the intensity matrix was built according to the Seurat object’s layout, with rows representing markers and columns representing cell types. Third, cell classifications were transformed into a one-column matrix labeled “Phenotype”, with each row containing the annotation for a single cell. Finally, the spatial coordinates of each cell were extrapolated from the QuPath data as “Centroid X Âµm” and “Centroid Y Âµm” and converted into new numeric vectors (x,y). The Seurat-like object was created from the SPIAT-formatted image intensity matrix using the “CreateSeuratObject” function. The matrix data was used in the *counts* slot of the new Seurat object. Subsequently, the cell classifications and the numeric x and y spatial coordinates were added to the object’s meta data. To enable spatial projection of cell types, the x and y coordinates were stored as dimensional-reduction embeddings in the *reductions* slot using the “CreateDimReducObject” function.

### STAT3D benchmarking

STAT3D computational efficiency and sensitivity were benchmarked against the Sopa [8] and Baysor [44] pipelines on the same processor. For Sopa, Spatial Data objects for TC70, KCP, and HCP were patched with a pixel width of 1900 and segmented per patch using 2D Cellpose with Sopa’s recommended nuclei diameter of 30 pixels. Tangram annotation was performed using the “HumanPrimaryCellAtlasData” reference dataset from the *celldex* package. Eventually, the annotated AnnData file was converted into a SingleCellExperiment object via the Bioconductor package *zellkonverter* and analyzed with RStudio libraries.

Baysor (v 0.7.1) benchmarking was performed after Windows Subsystem for Linux installation to ensure UbuntuOS access and Julia (v 1.11.1) download. The pipeline was run with the recommended configuration parameters such as segmentation in 2D with a 7.0 scale radius, 25% of scale standard deviation, and 10 as minimum molecule number per cell. The output Loom file (.loom), was processed in RStudio to extract the counts matrix, features (“Name”) and barcodes (“Cell ID”), stored in the *attrs* slot, which were required to generate a single cell experiment object compatible with Seurat libraries. The Baysor matrix, originally in “cell-by-gene” format, was transposed to “gene-by-cell” format and converted into a sparse matrix to conform to Seurat’s object standards.

### STAT3D functionality tests

The efficacy of the pipeline was tested across three datasets by comparing accuracy and assessing potential additive effects of its parameters (Table 4). First, to evaluate potential differences in STAT3D performance due to image reduction, three formats were analyzed: a single sharpest z-slice, the two sharpest z-slices, and the full z-stack. Second, 2D and 3D Cellpose segmentations were compared using the two z-slice stack formats. Third, STAT3D was run with a standard NE parameter (5 µm) suggested by 10x Genomics (https://www.10xgenomics.com/support/software/xenium-onboard-analysis), disregarding the STAT3D tailored calculation. Fourth, each dataset was analyzed regardless of STAT3D predictions for z-slice selection and NE. Here, STAT3D logic was tested by selecting random z-slices (“RZ”) or random nuclear expansions (“RNE”), while keeping the other STAT3D-derived measurement fixed. Lastly, the Sopa pipeline was run using STAT3D-derived nuclei diameter estimates (Table 5).

**Table 4.** STAT3D functionality-test settings

STAT3D tested settings	z-slices number in morphology.ome_STAT3D image	z-slice L-score	Cellpose segmentation	Example	Note
1_z_stack_2D	1	Highest	2D	KCP_1_Z_STACK_ 2D	
2_z_stack_2D	2	Two highest	2D	KCP_2_Z_STACK_ 2D	
2_z_stack_3D	2	Two highest	3D	KCP_2_Z_STACK_ 3D	
2_z_stack_3D_5µm_NE	2	Two highest	3D	KCP_2_Z_STACK_3D_5µm_NE	Pre-set NE at 5µm
All_z_stack_ 2D	All the z	Not accounted	2D	KCP_ALL_Z_STACK_2D	MIP satisfies only 2D segmentation criteria
All_z_stack_ 2D_RNE	All the z	Not accounted	2D	KCP_ALL_Z_STACK_2D_ RNE	Pre-set RNE
3_z_slice_2D_ RZ	1	Not accounted	2D	KCP_3_Z_SLICE_2D_ RZ	Pre-set not-STAT3D suggested z-slice number. Selected z-3.
SOPA_STAT3D_ DM	-	-	2D	KCP_SOPA_ STAT3D_ DM	Sopa pipeline run with diameter measurement (DM) derived from STAT3D calculations

**Table 5.** STAT3D and Sopa nuclei DMs

Dataset	STAT nuclei diameter size (microns /pixels)	Sopa nuclei diameter size (pixels)
KCP	7.6 /35	30
TC70	8.3 / 39	30
HCP	5.44 / 26	30

### Nondiseased dataset

The HD dataset exhibited features comparable to those of the HPC, TC70, and KCP datasets (3.3 GB, ∼8 millions of transcripts and ∼145 MB transcript.parquet file size). It was therefore included to validate gene expression analysis and to assess the accuracy of STAT3D in discriminating rare immune cell populations under nontumoral conditions.

## Results

### Validation strategy

The spatial transcriptomics analysis tool STAT3D was validated using four datasets, HCP, KCP, TC70, and HD, which differed in image sizes and number of transcripts (Table 6). A repertory of alternative set-ups (Table 4) was compared to 10x Genomics data without the STAT3D pipeline. The 10x Genomics data represented the study’s control data (“CTRL”) received by 10x Genomics and generated with Xenium on-board analysis platform with DAPI-nuclear cell segmentation. STAT3D was benchmarked against two other spatial transcriptomics pipelines Sopa [8] and Baysor [44].

**Table 6.** Datasets image size and transcript (parquet file) sizes

Dataset	Morphology.ome.tif size (MB)	Transcripts.parquet size (MB)	Number of transcripts
KCP	2246	89	5 115 684
TC70	2250	187	10 660 716
HCP	3515	492	28 455 659

### Tailored spatial transcriptomics pipeline for 2D and 3D analysis

STAT3D can be used to process images with tailored parameters measured within a standard Docker container, in a workflow that smoothly integrates diverse software and programming languages (e.g. QuPath, Cellpose, R, Python), enabling automated deployment of the pipeline applications in a lightweight container and the return of Seurat spatial objects compatible with standard community tools. STAT3D used Docker to automate the execution of the pipeline applications in a lightweight container and return one Seurat spatial object compatible with standard community tools. The workflow included an interactive selection of the level and the z-stacks to extract. Based on this architecture, STAT3D reduces image size by extracting single levels and in-focus z-stacks, creating a lighter and sharper image, while excluding lower-quality images from the original pyramidal OME-TIFF image (Fig. 1C). The quality of the new image was assessed using the L-score, which quantified the difference in sharpness across the z-stacks at chosen level (Fig. 1D). Next, STAT3D outputs one OME-TIFF image with single or multiple z-planes and launches QuPath for the calculation of image-based parameters such as nuclei diameter average and NE average, which were implemented respectively in the 3D Cellpose segmentation (with or without GPU support) and cell-to-transcripts mapping tasks. Subsequently, Cellpose masks were associated with cell transcripts in x, y, z or x and y coordinates according to the information stored in the “transcript.parquet” file. Eventually, STAT3D converted the data into a spatial Seurat object together with a spatial image in PDF format.

### NE with QuPath

Cytoplasmic transcripts can be misassigned to neighboring cells; therefore, STAT3D automatically calculated the optimal NE area for each cell and the average nuclei diameter. This process relied on QuPath’s measurements of the minimum average distance between nuclei and the average area of the nuclei.

### Generation of size-reduced and sharper 3D and 2D spatial transcriptomics images

Processing large image files without compromising single-cell and subcellular resolution remains a bottleneck in spatial omics. The capacity of spatial omics technologies to preserve tissue spatial heterogeneity results in images exceeding 2 GB, sometimes reaching up to 10 GB. This issue arises from the pyramidal structure of histological images, which contain morphological features at multiple resolution levels [45]. Several strategies, including gene decoupled multiscale feature extractors [46] and convolutional neural networks (e.g. Residual Network) [10, 47] have attempted to reduce computational demands and predict spatial gene expression by using tokens as basic vectors for representing and processing whole-slide image data. To address the issue of image size, STAT3D employed the *tifffile* library to extract only the highest-level image resolution, producing a smaller sized OME-TIFF image (Fig. 1A). For example, individual levels such as level 0, level 1, level 2, respectively, had image sizes of 3482 MB, 730 MB, and 19 MB, while the original image size was 2262 MB (Fig. 1B). Using the NumPy library, only two of the sharpest images from the z-stacks were automatically selected and merged into a new OME-TIFF image (Fig. 1C). STAT3D’s decision-making process over image levels and z-stack selection was guided by the L-score and configured via the user’s Snakemake *config.yaml* file. Overall, the highest L-scores were consistently seen in the same z-slices across all image levels (Fig. 1D and E). The L-score heatmap indicated that z-slices 4 and 5 at level 2 had the highest sharpness index in TC70 (Fig. 1E) and KCP (Supplementary CSV Table). Despite the high sharpness index calculated for the two KCP z-slices, the LS graph demonstrated a local maximum score for z-slice 4 (Fig. 1E). This result was attributed to a difference of 8977 in the sharpness index between z-4 (13349.9) and z-5 (4372.2) (Supplementary CSV Table). In HCP, z-5 and z-6 represented the sharpest slices (Fig. 1E). Due to HCP’s large dataset size, we additionally considered its MIP-derived image format, which resulted in a smaller image size of 23.14 MB. Ultimately, the resulting sharper and smaller STAT3D image showed an ∼98% reduction in size across all three data sets (Table 2). The image-analysis results revealed different scenarios associated with image size, thereby highlighting the importance of custom parameters to support each user’s decision-making process.

### Quality control metrics

The evaluation of quality control metrics in terms of blank codewords, negative control probes, and negative controls codewords in CTRL and STAT3D displayed values close to zero. Average values for Blank Codewords estimating background noise and false positive rates were 0.018 in STAT3D and 0.029 in CTRL pre-processed data. Negative controls measuring the binding of nonbiological sequences showed lower averages, with Control Probes at 0.009 and 0.006, and Control Codewords at 0.005 and 0.003 for STAT3D and CTRL, respectively (Supplementary Fig. S4).

### Image-tailored parameter measurements

The need for image-adapted spatial transcriptomics analysis represents a turning point in achieving customized, accurate single-cell mapping results. Diverse strategies focused on tailored data analysis, including novel models such as data clustering networks based on tailored spatial scale modulation [48] and computational probabilistic alignments between single-cell and spatial transcriptomics datasets to control the relative weight between transcriptional similarities and spatial graph distance [49]. Furthermore, the release of practical guidelines underlined strengths and weaknesses of sequencing-based or image-based technologies highlighting the need to provide flexible control over image resolution (e.g. “bin size” variation) and number of analyzed genes (e.g. “add-on” gene panel size) [50]. To address this need, STAT3D implemented single-cell parameter calculations using R on extracted QuPath image data (Fig. 1F). STAT3D used a heuristic calculation that estimated, in sequence, nuclei average area (“χ”, *A*), nuclei ray average (“σ”, *r*), centroids minimum distance average (“ψ”, *MCDA*), nuclei distance average (“ϴ”, *NDA*), and eventually nuclear distance (ϕ, *NE*) (Fig. 1F). STAT3D output all the measurements as a single Comma Separated Values File (.csv). The measured values were automatically implemented into the next steps of the pipeline. The heuristic STAT3D computational process resulted in image-based custom-tailored parameters at 3D single-cell resolution, where the NE values displayed confined coverage of nuclei areas with no overlap between nearby cells (Fig. 1A and B). Moreover, the STAT3D method achieved automated, customized cell parameter measurements without the need for complex unsupervised classification techniques.

### Image-based tailored cell segmentation

Cell segmentation involves drawing precise boundaries around cell perimeters or nuclei where imprecise performance can significantly impact downstream analysis. Image segmentation is necessary for *in-situ* sequencing-based and *in-situ* hybridization-based transcriptomics technologies, to enable the extraction of gene expression information and spatial context [51]. Nevertheless, the large size of spatial transcriptomics images and their encrypted data arrays resulted in prolonged segmentation times and massive RAM usage, demanding from 23 h [52] to ∼60 h [8] with memory peaks of 142 GB [52] and trillions of MB of data associated with segmented masks and transcripts locations [51]. Currently, multiple spatial transcriptomics platforms have been released with the aim of achieving accurate cell identification. These new methods offer convolutional network graphs to identify spatial domains with contiguous gene expression and histology [53], perform nuclear segmentation on hematoxylin and eosin (H&E) stained samples [54], semi-supervised segmentation frameworks based on single image region patching [55] and a Bayesian-based model that identifies cell types in spatial transcriptomics data and creates cellular maps [56]. However, improvements in segmentation performance time, the introduction of automated 3D resolution, and solutions for large output file sizes remained undisputed. The STAT3D pipeline addressed these bottlenecks by automatically performing image-tailored 3D or 2D spatial segmentation with the Cellpose *nuceli* model, taking advantage of the previously calculated average cell diameter value. In this context, the segmentation across different z-stack formats displayed reduced performance times, ranging from ∼3 to ∼100 min for 2D and 3D settings, respectively (Fig. 1h). The fastest segmentation was achieved with 2D STAT3D on the two sharpest z-slices (“STAT_2_Z_SLICE_2D”) and single sharpest z-slice (“STAT_1_Z_SLICE_2D”) settings, followed by the MIP-derived flattened 2D image (“STAT_ALL_Z_STACK_2D”) and the 3D segmentation of the two sharpest z-stack image (“STAT_2_Z_SLICE_3D”). The longest processing times for all datasets were observed with the Sopa per-patch segmentation approach (Fig. 1H). STAT3D expedited the cell segmentation step via GPU support through the NVIDIA Container Toolkit [57] integrated in the STAT3D container. The user’s GPU is automatically recognized when the NVIDIA drivers are correctly installed and configured on the user’s machine. The HCP image could not be processed at 3D resolution, as the tested NVIDIA RTX A500 GPU with only 4GB of VRAM did not provide sufficient memory to meet Cellpose 3D segmentation’s requirements. STAT3D stored the custom-tailored segmentation image together with the image masks in an OME-TIFF file. In the Toy dataset (55 MB) the CPU 3D segmentation runtime was completed in 19 min and 34 s, whereas GPU-accelerated segmentation finished in 1 min and 30 s.

STAT3D’s image-tailored segmentation accuracy was achieved by allowing decimal values for the customed “nuclei diameter” parameter, which Sopa did not support. Sopa pipeline based its analysis on Xenium-derived 2D flattened OME-TIFF formats (“morphology.focus.ome” and “morphology.mip.ome”) with a pixel size of 0.02125. Thus, STAT3D-derived measurements of 5.44 µm (HCP), 8.3 µm (TC70), and 7.6 µm (KCP) would correspond to 25.6-pixel, 39.05-pixel, and 35.76-pixels, respectively, rather than being rounded to integer values (Table 5). For the KCP dataset, rounding to 35 instead of 36 was imposed by the inability of the Sopa pipeline to accept a higher value such as 36. The Baysor method also disregarded the user-defined nuclei diameter. Consequently, the average expression of the immune cell targets did not significantly improve in any of the three datasets (Fig. 2).

**Figure 2.**
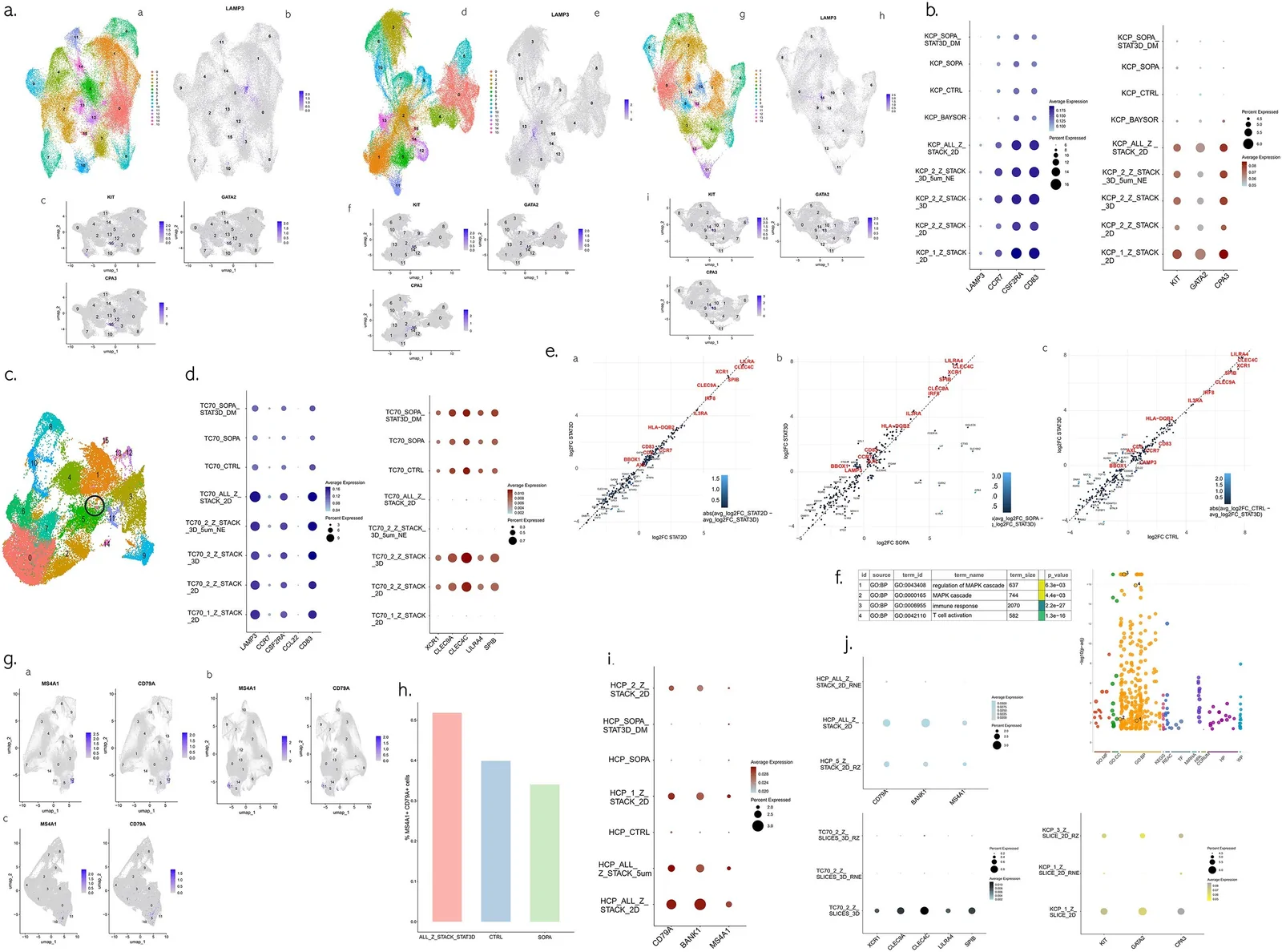
Comparative Analysis of spatial transcriptomics pipelines reveals improved immune cell identification with STAT3D approach. (**A**) Cluster-labeled UMAP projections of CTRL_STAT3D KCP (a), KCP_Baysor (d), and KCP_STAT3D_1Z_SLICE_2D (g). Feature plots highlighting LAMP3 transcript in CTRL_STAT3D KCP (b), KCP_Baysor (e), and KCP_STAT3D_1Z_SLICE_2D (h). Feature plots displaying expression levels of mast cell signatures (KIT/CPA3/GATA2) in CTRL_STAT3D KCP (c), KCP_Baysor (f), and KCP_STAT3D_1Z_SLICE_2D (i). (**B**) Dot plot displaying average expression levels and percentage of cells expressing LAMP3DC gene signature (LAMP3/CCR7/CSF2RA/CD83) (blue) and mast cell gene signatures (KIT/GATA2/CPA3) (red) in KCP dataset analyzed with nine different computational strategies. (**C**) Cluster-labeled UMAP projection of TC70 dataset analyzed with STAT3D after selection of the two sharpest z-slices of level two and segmented with 3D Cellpose segmentation. Myeloid-like cluster 14 enriched in cDC1s is highlighted with a black circle. (**D**) Dot plot displaying average expression levels and percentage of cells expressing LAMP3 DC gene signature (LAMP3/CCR7/CSF2RA/CD83) (blue) and DC [cDC1 and plasmacytoid dendritic cell (pDC)] gene signatures (XCR1/CLEC9A/SPIB/CLEC4C/LILRA4) (red) in TC70 dataset analyzed with eight different computational strategies. (**E**) Scatter plots displaying differential gene expression comparison between STAT3D versus STAT2D (a), STAT3D versus SOPA (b), and STAT3D versus CTRL in TC70 dataset. Each dot represents the log_2_ fold change (log_2_FC) value of a gene shared between the two methods. The dashed diagonal line (y = x) indicates equal expression changes in both conditions. Selected marker genes of interest (CLEC4C, LILRA4, SPIB, XCR1, CLEC9A, IRF8, IL3RA, BBOX1, HLA-DQB2, AXL, CD5, LAMP3, PCNA, CD83, CCR7) are labeled in red. (**F**) Manhattan plot displaying gene set enrichment analysis on significantly upregulated genes in Cluster 14 of STAT3D TC70 (avg_log_2_FC > 0.5, adjusted *P* <.05). Gene enrichment is conducted using g:Profiler (organism: Homo sapiens) with g:SCS multiple testing correction (significance threshold 0.05). The analysis includes annotated functional categories (e.g. GO terms, pathways, and other curated gene sets), highlighting biological processes and molecular functions significantly overrepresented among Cluster 14 marker genes. Only statistically significant enriched terms are shown. (**G**) Cluster-labeled feature plots of CTRL_HCP (a), ALL_Z_STACK_STAT3D_HCP (c), and SOPA_HCP highlighting B cell gene transcripts (MS4A1/CD79A) expression patterns. (**H**) Bar plots showing the percentage of cells double positive for B cell signatures (MS4A1/CD79A) in HCP dataset analyzed with three different methods (ALL_Z-STACK STAT3D HCP, CTRL_HCP, and SOPA_HCP). (**I**) Dot plot displaying average expression levels and percentage of cells expressing B cell gene signature (CD79A/BANK1/MS4A1) in HCP dataset analyzed with seven different computational strategies. (**J**) Dot plots displaying STAT efficient identification of rare immune cells in HCP (a), KCP (b), and TC70 (c) datasets in comparison to partially insensitive STAT pipeline setup (Random nuclear expansion RNE, Random Z-slice selection RZ).

STAT3D allowed full control over the pipeline’s outputs. Firstly, the “export” folder encapsulated all images of the z-stacks at the chosen level, facilitating a deeper understanding of image architecture and aiding decision-making for subsequent steps (e.g. z_top1 and z_top2, one z-stack, all z-stack selection). Second, the visual inspection of images was quantitatively supported by the automatic L-score calculation, which evaluated features by their locality preserving power [58]. This metric helped identify the crispest z-stacks and assess image quality (Fig. 1). Notably, the L-scores revealed a clear trend in image sharpness: within each level, the same z-stack numbers consistently showed the highest index and were often located in the middle of the z-stack (Fig. 1E and Supplementary CSV Table). TC70 and KCP pyramidal images contained eleven z-stacks (Supplementary Tables S1 and S2), with z-4 and z-5 being the sharpest slices (Fig. 1E and Supplementary CSV Table). However, the L-score projection of the smaller datasets showed a unimodal trend centered on slice z-4 (Fig. 2 and Table 6). Conversely, the HCP image comprised 13 z-stacks (Supplementary Table S3), within high L-scores for z-5 and z-6 (Supplementary CSV Table). As the largest dataset among the three, HCP showed better sensitivity after MIP (Fig. 2I), suggesting that MIP better captures the signal across the 13 z-slices. Furthermore, the MIP option enabled processing of the largest dataset on a machine with 32 GB of RAM. MIP is the user-selectable parameters, allowing exploration of larger datasets (>2.3 GB), overcoming processor’s “Out Of Memory” (OOM) limitations. In all datasets, inspection of every z-stack image revealed fragmented images, often appearing as the first or last images of the stack (Supplementary Figs S8–S10). The final STAT3D outputs exhibited reduced image sizes not achieved by other methods, leading to smaller downstream outputs (Fig. 2J) and more efficient and standardized data processing, thereby reinforcing data robustness. In addition, spatial crowding between T and B cells quantified with FNN package was moderate for STAT3D (∼8%), negligible for Sopa (∼0.2%), and substantially higher for CTRL (∼15%) (Supplementary Fig. S12). Overall, we demonstrate that the STAT3D workflow is flexible and adaptable to different user file sizes.

### Reduced Seurat spatial object dimension

Spatial transcriptomics pre-processing files, such as spatial *DimReduc* Seurat objects, can hinder downstream data analysis by reaching thousands of MB in size and by slowing the computational process [59]. This issue has not been addressed yet by the spatial omics community. Here, STAT3D offered a solution by re-framing the spatial data via cell-to-transcript mapping and generating a new spatially reduced Seurat object. The cell-to-transcript mapping step merged Cellpose segmentation information from the NumPy array (.seg.npy) with the 3D spatial localization of the transcripts stored in *transcripts.parquet* file. This method resulted in lighter barcodes.tsv, features.tsv and matrix.mtx files, which formed the basis of the new STAT3D gene expression matrix (Table 7). With this strategy, STAT3D freed users from tangential heavy archive files such as gzipped tape archives (.tar.gz) (e.g. *analysis.tar.gz* of ∼ 10 000 KB) or ZEMAX archive (.zarr) (e.g. *cells.zarr.gz* of ∼ 78 000 KB). These archive files contained only raw data, intermediate files, and analysis results from the Xenium Onboard Analysis software. To validate STAT3D output, we compared the difference in file size of the Seurat DimReduc object across all the validated STAT3D settings, the CTRL data, Sopa and Baysor files. (Fig. 1I). STAT3D spatial objects were -the smallest ranging between 700–1000 MB in HCP, 200 MB in KCP, and 300 MB in TC70, approximately (Fig. 1I). Conversely, Sopa, Baysor, and CTRL files reached sizes approximately twofold to threefold higher than STAT3D (Fig. 1I). Baysor data analysis was performed only in KCP due to its intrinsic limitation for big-data handling. We demonstrated that STAT3D effectively addressed large spatial transcriptomics file sizes and contributed to downstream analysis improvement.

**Table 7.** Examples of feature.tsv, barcodes.tsv and matrix.mtx file sizes with and without pre-processing with STAT3D pipeline

	Dataset	Barcodes.tsv.gz size (KB)	Features.tsv.gz size (KB)	Matrix.mtx.gz size (KB)
Pre-processing with STAT3D	KCP_1_z_stack_2D	187	4	4504
Pre-processing without STAT3D	CTRL_KPC	248	5	10 355
Pre-processing with STAT3D	TC70_2_z_stack_3D	253	4	5775
Pre-processing without STAT3D	CTRL_TC70	300	5	13 487
Pre-processing with STAT3D	HCP_All_z_stack_ 2D	549	4	22 553
Pre-processing without STAT3D	CTRL_HCP	782	5	52 426

### High resolution of immune cell populations

To comprehensively and objectively characterize the TME landscape, it is essential to analyze immune cell composition, their spatial distribution, and their interactions with neighboring cells. This spatial omics approach can help elucidate the complex interplay between tumor and immune cells and provide a foundation for identifying reliable biomarkers [60, 61]. To validate STAT3D accuracy in the discrimination of lymphoid and myeloid immune cell subsets in the TME and in a nontumoral context, we used the unsupervised nonlinear dimensionality reduction approach.

Compared with CTRL, Sopa, and Baysor, STAT3D data showed improved cell clustering for the discrimination of LAMP3^+^ DCs (LAMP3/CCR7/CD83/CSF2RA) and mast cells (KIT/GATA2/CPA3) in the KCP dataset (Fig. 2A). Both the average gene expression levels and the proportions of cells expressing immune cell signatures were enhanced in STAT3D-derived data (Fig. 2B). Differential gene expression analysis of the KCP data set grouped LAMP3, CCR7, CD83, and CSF2RA genes into a DCs-specific cluster only in STAT3D pre-processed data (Fig. 2A and Supplementary Fig. S5). By contrast, in CTRL data, LAMP3 was found among the most significant genes (average log_2_FoldChange > 1) of the heterogeneous cluster 3, together with other genes (e.g. AMY2A and CYP2A7) not associated with canonical LAMP3^+^ DC gene signatures (Fig. 2A and Supplementary Fig. S5). Similarly, Baysor clustering placed LAMP3^+^ DCs within the heterogeneous cluster 2 (Fig. 2A and Supplementary Fig. S5).

STAT3D, CTRL, and Sopa identified a specific DCs-like cluster in the TC70 dataset. In the STAT3D *2 z-stack 3D* setting, this population was detected in cluster 14 (Fig. 1C). In contrast, in the STAT3D *2 z-stack 2D* setting, CTRL and Sopa, the gene signatures associated with DCs-like populations were located in clusters 13, 14, and 12, respectively (Supplementary Fig. S6). cDC1s (XCR1/CLEC9A/SPIB) and pDCs gene signatures (LILRA4/CLEC4C) were consistent in both the *2 z-stack 3D* and *2 z-stack 2D* STAT3D settings (Fig. 2D). Notably, only the *2 z-stack 3D* analysis revealed statistically significant changes in BBOX1 expressions (Fig. 2E). Consistently, the Biological Process subontology of g:GOSt in *2 z-stack 3D* cluster 14 highlighted gene products involved in the MAPK cascade, immune response, and T cell activation networks (Fig. 2F). In the HD dataset, STAT3D robustly inferred a higher fraction of cells expressing macrophages-associated markers (MRC1 and CD68; 5%–10%), whereas the fraction of marker-positive cells accounted for less than 2% in Sopa and CTRL-derived datasets (Supplementary Fig. S14A).

We interrogated the HCP dataset for immune cell populations, focusing on B cells (CD79A/MS4A1), using different assay features of the Seurat spatial object (e.g. gene identity) (Fig. 2G). Transcripts associated with B cells were localized primarily to cluster 12, 11, and 5 for Xenium (CTRL), Sopa, and STAT3D, respectively, in the *All z-stack 2D* setting (Fig. 2G). The highest proportion of CD79A/MS4A1 double-positive cells, ∼0.55% of the total cell population, was detected with the STAT3D method, followed by CTRL (∼0.4%) and Sopa (∼0.35%) (Fig. 2H). STAT3D showed with the greatest sensitivity for detecting B cell-associated transcripts (CD79A/BANK1/MS4A1), yielding the highest mean expression levels and the largest fraction of expressing cells (Fig. 2I). Consistently, SingleR annotation using the “HumanPrimaryCellAtlas” reference in HCP and TC70, mirrored the manual annotation and listed B cells and DCs among the cell types with the strongest prediction scores. Similarly, the automated annotation of KCP using the “NovershternHematopoieticData” reference assigned high prediction scores to DCs and granulocytes (Supplementary Fig. S7). The confidence of the comparison between expression profiles and reference datasets was consistent with the gene expression levels observed with the STAT3D pipeline for the three target cell types.

With STAT3D, we improved cluster separation and identified significant population-specific genes for the lymphoid and myeloid populations, not detectable with standard Xenium pre-processed data or via alternative spatial transcriptomics pipelines.

### Logic robustness of STAT3D

To evaluate the ability of STAT3D features to identify rare cell populations such as B cells, LAMP3^+^ DCs and mast cells, across different tissue types, we performed functionality tests. First, we ran the Sopa pipeline using STAT3D’s DMs (Tables 4 and 5, and Supplementary Fig. S13E). This resulted in a marginal improvement in the average gene expression level in the TC70 dataset (Fig. 2D), HCP dataset (Fig. 2I), and HD dataset (Supplementary Fig. S13A), although the effect was less pronounced than with STAT3D levels. Second, we analyzed the STAT3D pipeline at *2 z-stacks in 3D* with a preset NE of 5 µm following standard 10x Genomics procedures. This test produced variation in the KCP and HD datasets due to its close similarity to the STAT3D-measured NE (5.19 and 6.6 µm, respectively) (Fig. 2B and Supplementary Fig. S13A). In contrast, in TC70, the average gene expression of cDC1 gene signatures was close to zero (0.002) with fewer than 0.3% of cells detected (Fig. 2D). The pre-defined NE implemented in the HCP dataset also showed poorer performance compared to the tailored STAT3D measurements (Fig. 2I). Lastly, we tested the STAT3D pipeline using the nominally optimal setting for each dataset: *1 z-stack 2D* for KCP, *2 z-stack 3D* for TC70, and *All z-stack 2D* for HCP and HD, without the automated logic of STAT3D (Table 8 and Supplementary Fig. S13F). In all four datasets, random selection of the z-stack or random assignment of the NE parameter yielded suboptimal downstream data, as evidenced by reduced detection of B cells, mast cells, DC gene signatures (Fig. 2j and Supplementary Fig. S13B), and out-of-focus poor spatial resolution (Supplementary Fig. S13C and D). The outcome of this automated logic test was attributed exclusively to these stochastic selections, as all other automated parameters were kept identical to those used in the standard automated run.

**Table 8.** STAT3D automated logic test

Dataset	STAT3D suggested z-selection	Random z-selection	STAT3D suggested NE (µm)	RNE (µm)
KCP	z 4	z 3	6.4	2
TC70	z 4_z5	z 3_z6	3.9	10
HCP	All z-stack	z 5	7.89	2

### Immune cells spatial projection

STAT3D-identified immune cell types were projected into a lower dimensional space using spatial UMAP from the Seurat package (Fig. 3). In the KCP dataset, LAMP3^+^ DCs co-localized within a specific region enriched in CD4^+^ T cells and B cells (Fig. 3), whereas mast cells showed a scattered distribution throughout the entire tissue biopsy without forming nucleated aggregates. Regions enriched in LAMP3⁺ DCs exhibited increased staining intensity in the KCP-derived H&E image (Fig. 3A). In the TC70 spatial UMAP projection, LAMP3^+^ DCs co-localized with CD8^+^ (CD3E/CD8) and CD4^+^ T (CD3E/CD4) cells, forming several small islets at the tumor biopsy margins. In the central region of the tumor biopsy, cDC1s (ITGAX/XCR1/CLEC9A) clustered within neighborhoods containing other immune cells, including B cells (MS4A1/CD79A), CD8^+^ (CD3E/CD8), CD4^+^ (CD3E/CD4), and T cells (Fig. 3B). H&E staining of TC70 showed LAMP3⁺ DCs-enriched regions localized within tumor-like nests, while cDC1s infiltrated the central tumor areas in close association with T lymphocytes-dense regions (Fig. 3B). In the HCP dataset, B cells were located at the tissue periphery, in proximity to CD4^+^ T cells. Here, the H&E image showed B cells specifically in areas surrounding adipocyte-like aggregates (Fig. 3C). Euclidean pairwise distance analysis using SingleR labels in HCP, highlighted the spatial separation of B cells from other immune cell types (Fig. 3D), mirroring the pattern observed in the spatial UMAP projection and the H&E staining.

**Figure 3.**
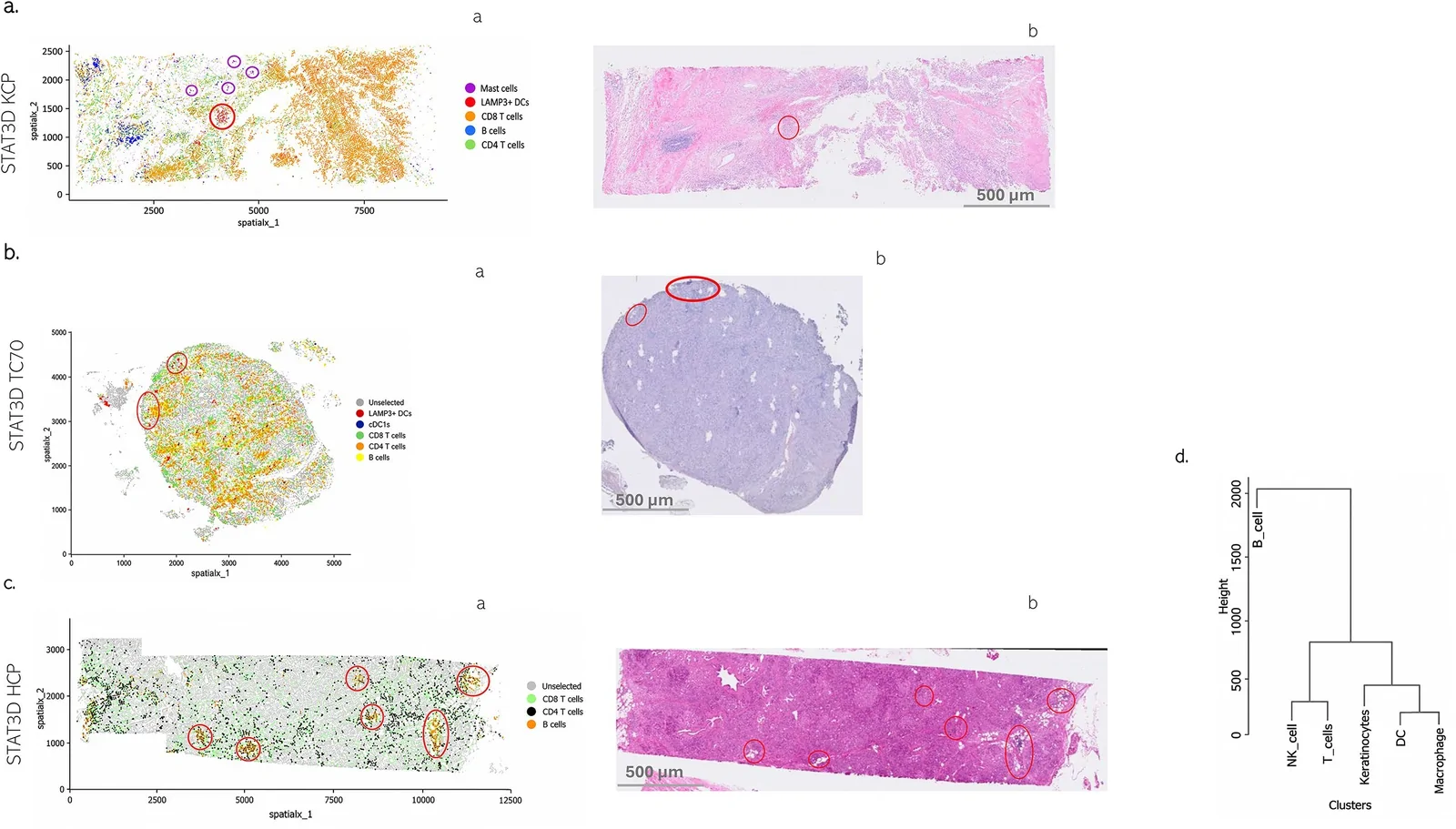
STAT3D spatial UMAPs reveal immune cell organization and tissue localization across KCP, TC70, and HCP datasets. (**A**) Spatial UMAP of KCP dataset generated with STAT3D workflow. Mast cells (CPA3/KIT/GATA2), LAMP3 DCs (LAMP3/CCR7/CD83/CSF2RA), CD8^+^ T cells (CD8A/CD3E), CD4^+^ T cells (CD4/CD3E), and B cells (MS4A1/CD79A/BANK1) are respectively highlighted in purple, red, orange, light green, and blue dots. H&E staining (by 10x Genomics) of KCP dataset-derived tissue (b). The area enriched in LAMP3 DCs is highlighted in red (a and b). Mast cells are highlighted with purple circles (a). (**B**) Spatial UMAP of TC70 dataset generated with STAT3D workflow. cDC1s (XCR1/CLEC9A/ITGAX), LAMP3 DCs (LAMP3/CCR7/CD83/CSF2RA), CD8^+^ T cells (CD8A/CD3E), CD4^+^ T cells (CD4/CD3E), and B cells (MS4A1/CD79A/BANK1) are respectively highlighted in red, black, light green, orange, and yellow dots. QuPath-derived H&E projection of TC70 dataset-derived tissue (b). The tumor nest areas enriched in LAMP3 DCs and cDC1s are highlighted in red and black respectively (a and b). (**C**) Spatial UMAP of HCP dataset generated with STAT3D workflow. CD8^+^ T cells (CD8A/CD3E), CD4^+^ T cells (CD4/CD3E), and B cells (MS4A1/CD79A/BANK1) are respectively highlighted in light green, black, and orange dots. H&E staining (by 10x Genomics) of HCP dataset-derived tissue (b). The areas enriched in B cells are highlighted in red (a and b). (**D**) Dendrogram displaying the spatial Euclidean distances with hierarchical clustering using SingleR annotation labels in HCP dataset.

### High resolution of LAMP3^+^ DCs cluster in TC70 dataset

Spatial transcriptomics technologies enable the localization of gene transcripts at single-cell, subcellular resolution while preserving tissue integrity [62]. The ability to study the relationships among spatial architecture, cellular states, and interactions is pivotal to understanding TME organization. Currently, investigations of the myeloid immune compartment, particularly for rare cell populations such as DCs, remain challenging due to technological limitations, including gene panel size, lack of specific biomarkers that selectively discriminate between cell types, and the low number of target cells. With our method, we first demonstrated that the STAT3D pipeline can enable accurate localization of spatial neighborhoods of immune cells (Fig. 3). We further verified the STAT3D prediction of LAMP3^+^ DCs in TC70 with five-plex immunofluorescence microscopy (Fig. 4A and B). The mIF panel targeting LAMP3, CCR7, IDO1, CD8, and Cytokeratin (PanCK) enabled the identification of two regions of interest (ROIA and ROIB, Fig. 4A) characterized by co-localization of antibody staining at high fluorescence intensities. Phenotypic analysis of the cells, performed using SPIAT-formatted intensity data, identified 10 cell types and 12 immune-infiltrated regions (Fig. 4B and Supplementary Table S4). Here, unsupervised clustering resolved six main cell clusters (Fig. 4C), which were spatially reconstructed into the mIF ROIs (Fig. 4C). Integrating SPIAT phenotyping with the Seurat clusters revealed the spatial architecture and cell proportions in ROIA and ROIB. LAMP3^+^DCs (“CCR7/LAMP3 cells”) were assigned to cluster 4, and localized at the core of both ROIs, in close proximity to cluster 5, which consisted mainly of CD8^+^ T cells and IDO1^+^LAMP3^+^ DCs and was surrounded by tumor cells (“PanCK cells” and “PanCK/IDO1 cells”) (Fig. 4D). The presence of a nest-shaped area of PanCK^+^ cells encompassing regions enriched in LAMP3^+^ DCs and CD8^+^ T cells was quantified using the cross-K function and visualized relative to the Poisson distribution (Fig. 4E). The black line representing LAMP3^+^ DCs and CD8^+^ T cells distribution crossed the randomly distributed points generated with the Poisson point process (red dotted line) at ∼44% of the specified distance (100) in the cross-K function, indicating a nonrandom spatial pattern and confirming the nest-shaped organization of tumor predicted by the STAT3D pipeline. Both ROIs were enriched in LAMP3^+^ DCs cells and displayed neighboring regions of CD8^+^ T cells (Fig. 4C and D). NMS analysis was used to assess the spatial proximity among the different ROI regions and cell types (Fig. 4F). NMS revealed stronger interactions between LAMP3^+^ cells and CD8^+^ cells (score 0.47), whereas lower normalized mixing scores were detected between the PanCK/CD8 region and IDO1^+^cells (0.20) or PanCK/CD8 region and CCR7^+^ cells (0.21) (Fig. 4F). STAT3D showed higher sensitivity for identifying single cells than the Xenium Explorer tool in ROIA and ROIB (Fig. 4G) and in the KCP dataset (Supplementary Fig. S11). The proportions of neighboring cells, including CD8⁺ T cells and DCs, differed between CTRL and STAT3D methods. In the two ROIs, CD8⁺ T cells comprised 10.9% of neighboring cells, while DCs accounted for 0.36% in CTRL TC70 samples. In contrast, in STAT3D (*2 z-stack 3D*) data, CD8⁺ T cells and DCs accounted for 7.72% and 0.38% of neighboring cells, respectively. Taken together, STAT3D demonstrated its reliability in helping depict the immune compartment’s architecture in relation to tumor-infiltrated regions.

**Figure 4.**
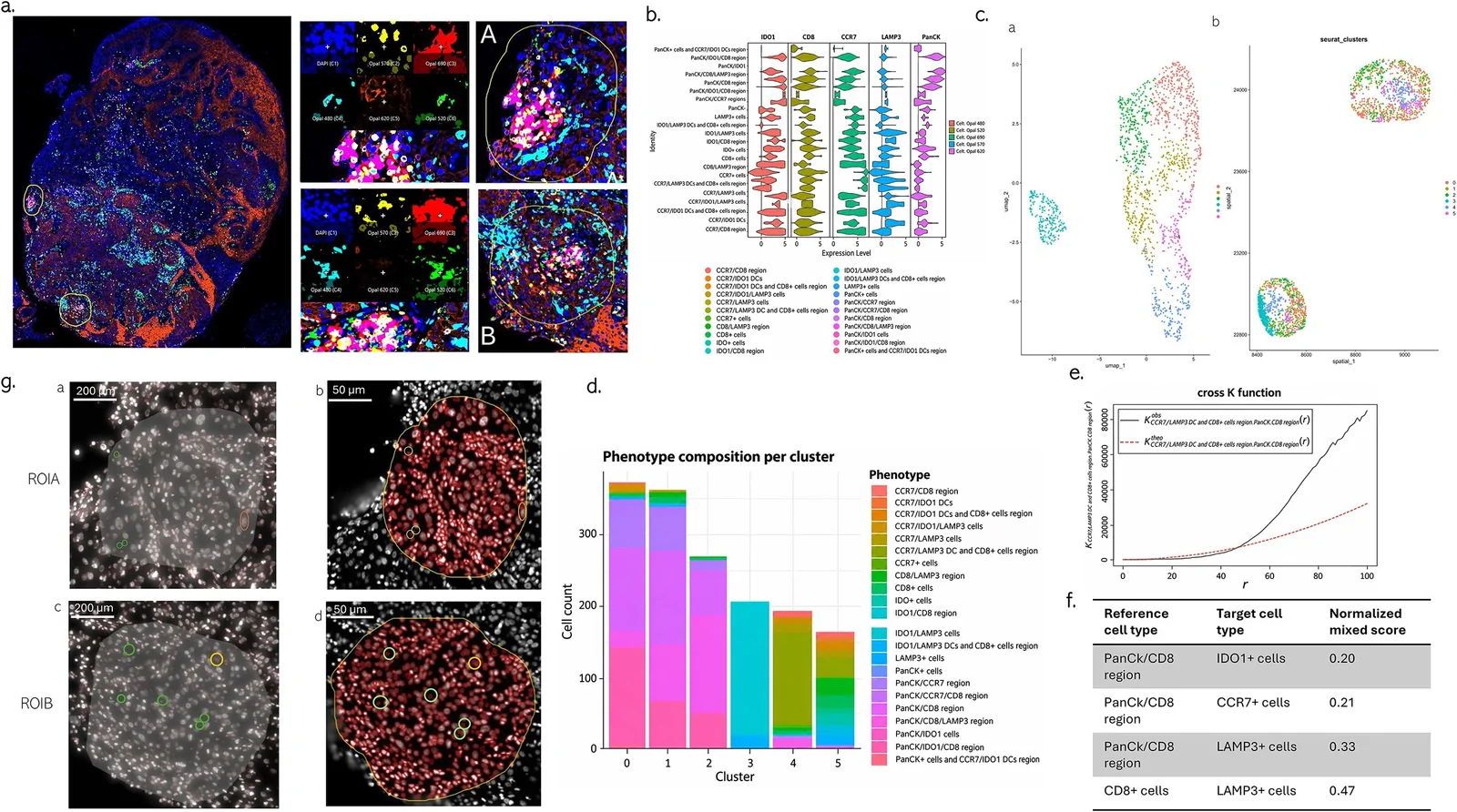
mIF and STAT3D-based image analysis identify and quantify immune cell regions in TC70 tumor biopsy. (**A**) Immunofluorescence images of TC70 tumor biopsy. LAMP3^+^, CCR7^+^, CD8^+^, IDO1^+^ enriched regions (A and B) are highlighted in yellow circles. (**B**) Violin plots displaying the distribution of expression levels for the five-plex mIF markers (IDO1/CD8/CCR7/LAMP3/PanCK) in each cell type or cell region identified with the mIF assay in ROIA and ROIB. (**C**) UMAP based on mIF-derived mean marker intensities displaying six cell clusters distributed across the two ROIs. Spatial UMAP projection of the six mIF-derived Seurat clusters in ROIA and ROIB. (**D**) Stacked box plot displaying the composition of the six mIF-derived Seurat clusters in terms of mIF phenotypes (cell types and cell regions). (**E**) Cross-K function plot displaying the co-localization of the target region “CCR7/LAMP3 DCs and CD8^+^ cells region” relative to the reference cell region “PanCK/CD8^+^ cells region”. Cross-K values are shown on the *y*-axis for a maximum radius of 100 on the *x*-axis. Randomly distributed points are displayed using a Poisson point process (red dashed line). (**F**) Table displaying the normalized mixing score (NMS) in the two ROIs considering four tumor–immune cell spatial interactions. (**G**) Image analysis in ROIA and ROIB in the TC70 dataset displaying low-intensity regions identified with Xenium Explorer software under CTRL settings (a, c) and QuPath Image Software using STAT3D-tailored parameters (b, d). Green circles indicate discrepancies in cell detection, while orange circles indicate differences in cell segmentation.

## Discussion

Spatial omics technologies open new avenues for investigating biological questions at single-cell and sub-cellular levels. Yet, standardizing and expediting data pre-processing, while maintaining image uniqueness, remains a bottleneck for the field. To address these challenges, we developed STAT3D, a user-friendly and customizable spatial transcriptomics workflow. STAT3D streamlines processing time and reduces file sizes while delivering nonconventional 3D cellular segmentation, all without sacrificing tissue sub-cellular detail. We present a fully enclosed Docker tool designed to minimize computational effort, enabling researchers from diverse scientific backgrounds and with varying levels of bioinformatics expertise to use the platform efficiently with regular laptops and high-performance clusters.

STAT3D expedites cells annotation with SingleR, which is considered the most optimal reference-based method for Xenium-derived data [63]. Among the benchmarked pipelines, only STAT3D provides automatic annotation without requiring manual data conversion by the user (e.g. from AnnData to SingleCellExperiment) or the laborious search for compatible reference datasets. Quality control metrics analysis demonstrates that STAT3D achieves high assay specificity and decoding accuracy, with zero or near-zero counts across all control categories. As spatial transcriptomics imaging datasets grow, leading to greater computational demands and longer processing times, STAT3D integrates GPU acceleration for 2D and 3D cell segmentation steps. Nevertheless, to address GPU constraints related to limited NVIDIA VRAM, STAT3D provides an automated and customizable workflow for 3D Cellpose nuclei segmentation in CPU-only mode. STAT3D substantially reduces segmentation times compared with the other pipelines and stands out by including decimal values for its parameters, avoiding approximations and forced data alteration. This highlights STAT3D’s flexible and user-oriented design. Furthermore, we demonstrate that the diameter parameter alone is insufficient to increase Sopa’s sensitivity, marking the importance of every STAT3D feature and its automated logic.

We observed that the smallest dataset such as KCP displayed a LS peak-trend. Consequently, the data generated by STAT3D at *1 z-stack 2D* in KCP exhibited higher accuracy across the different functional tests. However, TC70 and HCP did not follow a peak distribution, suggesting high variability across images and emphasizing the importance of STAT3D’s detailed image information. Thus, in the absence of a distinct peak, when two z-slices exhibit comparable sharpness, we suggest automated STAT3D analysis using selection of two z-slices followed by 3D cellular segmentation. Nevertheless, due to computational constraints, large datasets may alternatively be analyzed using MIP. We speculate that the superior performance of *2 z-stack 3D* over *2 z-stack 2D* is due to mixed data processing: the multiplane format (2-zeta) is processed with a 2D segmentation approach rather than with the 3D, which may reduce segmentation accuracy for cells located in the lower z-slices or in the background.

The STAT3D workflow prevents cell-transcript mislabeling and accurately predicts the trends of contiguous immune cell frequencies later confirmed with mIF. The risk of transcripts mislabeling for cells in close contiguity is greater and related to sharp cells’ detection. Discrepancies in cells identification can be avoided with STAT3D workflow due to the flexibility of the pipeline, which allows user’s customization through parameters such as threshold intensity and noise reduction. Furthermore, FNN equations highlight the capability of STAT3D to quantify cellular interactions at short spatial distances (20 radii). Here, STAT3D data suggest moderate crowding among cell types that frequently interact in the TME and give rise to cell aggregates such as tertiary lymphoid structures [12].

Although STAT3D overcomes major limitations in spatial transcriptomics, it also highlights a new challenge. Specifically, it underscores the need for flexible tailoring of heterogeneous tissue biopsies that contain both low- and high-cell-densities regions. In such cases, applying a single NE parameter based on average cell values may constitute a seemingly tailored but ultimately confounding factor.

Furthermore, functional tests across all datasets reveal the importance of STAT3D automated logic for the successful detection of key transcripts associated with rare myeloid and lymphoid cell types. In addition, STAT3D could identify gene transcripts involved in signaling pathways associated with tumor progression such as epithelial to mesenchymal transition (ETM) in TC70 dataset. Notably, ETM transition in head and neck cancer subtypes has been documented in previous studies [64–66], in line with reports implicating receptor tyrosine kinase pathways and their downstream effectors (e.g. MAPK and PI3K) [67].

The spatial projections of KCP’s cell types with STAT3D localize LAMP3^+^ DCs in proximity to B cells and CD4^+^ T cells suggest the formation of tertiary lymphoid structures [12]. Interestingly, STAT3D’s observation of sparse mast cells in KCP-derived data is supported by a previous study [68], which reports their presence at low frequencies and highlights their role in tumor angiogenesis. Moreover, STAT3D immune cell projections in TC70 suggest a tumor-suppressive microenvironment characterized by sustained immune cell crosstalk between cDC1s and CD8^+^ T cells, possibly driven by established ligand-receptor interactions such as XCR1-XCL1 or CXCL9/10–CXCR3 [69]. Remarkably, the STAT3D workflow detects B cell clusters in the HCP dataset in a strong relationship with adipocyte-rich areas, in alignment with current scientific discoveries elucidating the metabolic crosstalk among immune cells, cancer-associated adipocytes, and fibroblasts in the TME of pancreatic ductal adenocarcinoma [70]. B-cell niches are hypothesized by Sopa only after overlapping H&E staining with the spatial transcriptomics data [8]. This demonstrates STAT3D’s superior sensitivity and its ability to predict rare immune cell types in dense tumor tissue biopsies such as pancreatic adenocarcinoma, without requiring H&E data integration. STAT3D improves spatial cell annotation of highlyplastic and heterogeneous cell types in HD dataset. Notably, under nontumoral conditions, STAT3D recognizes genesignatures characteristic of tissue-resident macrophages, including MRC1, consistent with the findings reported by Cheung *et al*. [68]. Through its automated heuristic calculations, STAT3D enables annotation of rare myeloid cell types such as LAMP3^+^ DCs in the KCP and HD datasets. The identification of this cell type is consistent with Zhang *et al*.’s single-cells tudy, which suggests that LAMP3^+^ DCs are involved in lymphocyte regulation during hepatocellular carcinoma development [71].

By using STAT3D pre-processed data, we explored novel networks within small immune cell compartments and examined the connections between cellular states and spatial localization across different tumoral and physiological landscapes. Our study demonstrates that STAT3D serves as a versatile, user-oriented, and fully automated computational pipeline that overcomes challenges in current spatial transcriptomics approaches. With its capacity to provide reliable and high-resolution outputs, STAT3D enhances biological insights and opens new opportunities for major discoveries.

## Limitations

STAT3D was developed using a dataset that reached a maximum size of 3.4 GB and ∼28 million transcripts. Large datasets generated with wider tissue imaging approaches, such as tissue microarrays (TMAs), can reach file sizes of up to 18 GB, potentially compromising STAT3D performance and data handling. To overcome this, STAT3D repository includes a “scalability” section with a library-downgrading example to support large TMAs file processing while maintaining the pipeline efficiency. In addition, to ameliorate time-intensive runs, the increase of computational resources such as powerful GPUs with ∼300 nodes, greater processors (e.g. 4 × NVIDIA GH200 Grace Hopper Superchips per node with 72-core ARM CPU paired with GPU), larger memory space (e.g. 96 GB HBM3 and 128 GB LPDDR5 per Grace Hopper module) and more accommodating local storages (e.g. 1.92 TB local NVMe SSD on each node) can further improve STAT3D performance.

## Recommendations

The image may first be assessed with image-analysis software such as QuPath for to determine the optimal intensity threshold to specify in STAT3D’s configuration file. For large datasets, such as HCP, we recommend using the “*All z-stack 2D*” approach. For smaller datasets, we suggest examining the LS CSV file generated by STAT3D to identify potential score peaks.

## Supplementary Material

gkag937_Supplemental_Files

## Data Availability

The code developed in this work is available in FigShare (doi: https://doi.org/10.6084/m9.figshare.33456808). The Apptainer files (.sif and .tar) are available in FigShare (doi: https://doi.org/10.6084/m9.figshare.33461998).
