# Supplementary material for: STAT3D as a new image-tailored Xenium-based spatial transcriptomics tool for accurately dissecting the tumor microenvironment
Source: Nucleic Acids Res. 2026 Oct 8;54(18):gkag937. doi: 10.1093/nar/gkag937 (PMC13646825; doi:10.1093/nar/gkag937)
Supplement: Supplementary file 1 [file gkag937_supplemental_files.zip › Supplementary-data-review-R2.pdf]

Supplementary Code

The directed acyclic graph (DAG) workflow of the Snakemake pipeline can be generated and rendered as a PNG using Graphviz in a single command. To ensure proper parsing, the first line of configuration metadata is skipped.

Users should adjust the path to their local configuration file as appropriate and the path in the config_toy_dataset_2D_CPU_DAG.yaml file.

The configuration file for DAG test is located at:

stat3d/configs/config_toy_dataset_2D_CPU_DAG.

DAG generation command:

snakemake --configfile /path/to/ config_toy_dataset_2D_CPU_DAG.yaml --rulegraph 2>snakemake.log | Select-Object -Skip 1 | dot -Tpng -o workflow.png

Workflow DAG visualization

The resulting PNG file (*workflow.png*) displays the DAG of all Snakemake rules and their dependencies.

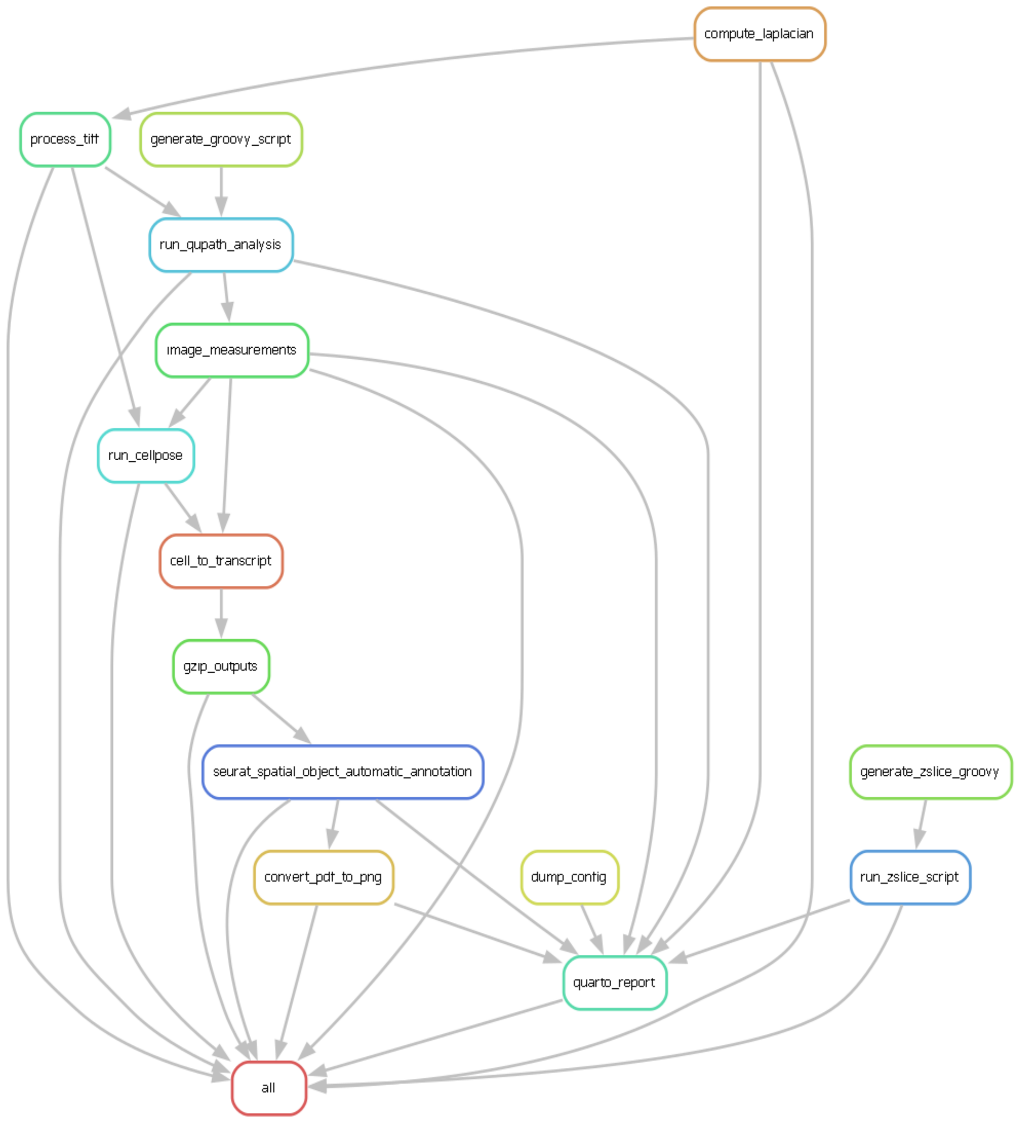

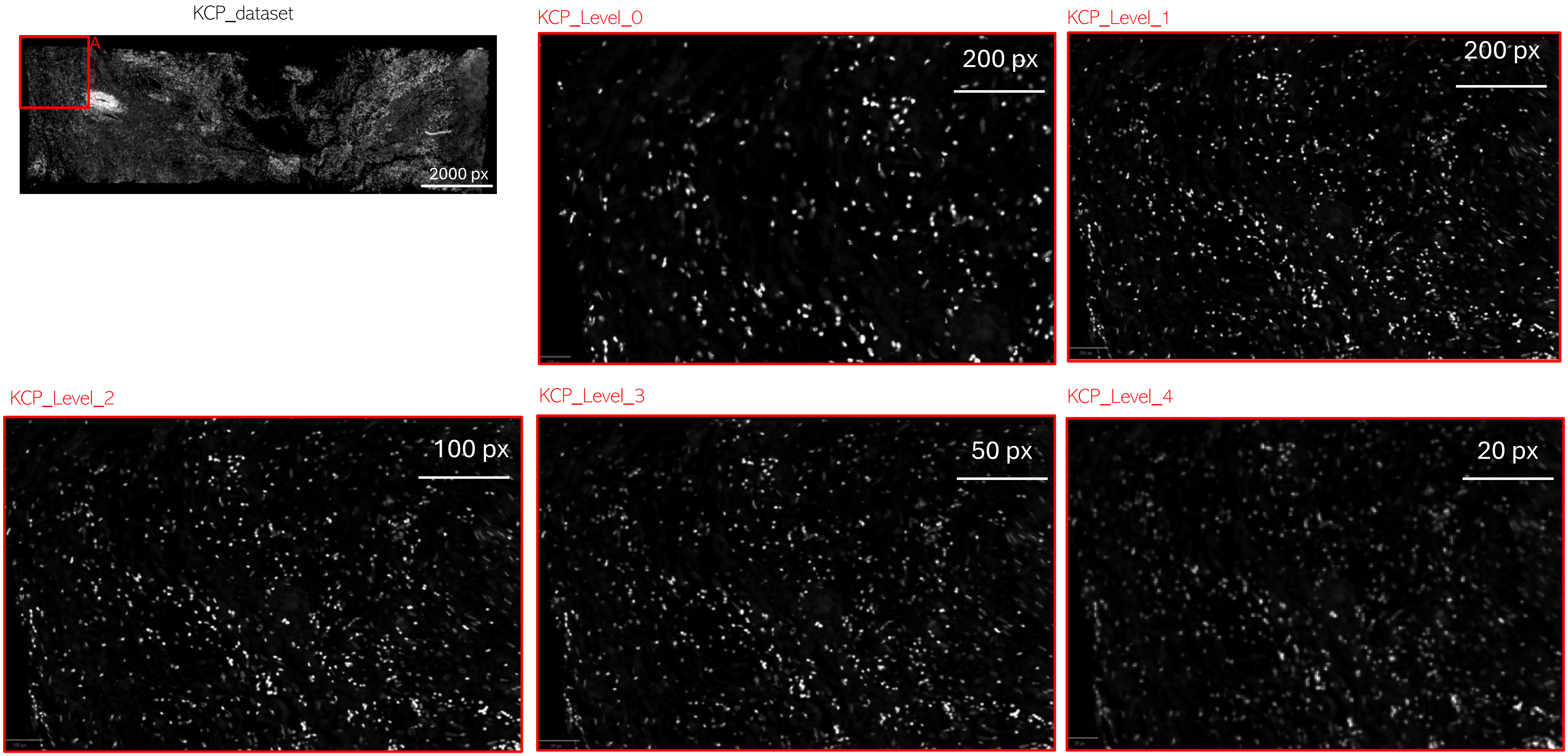

Supplementary Figure 1. Levels resolution in KCP dataset.
KCP dataset images derived from four different level (0,1,2,3,4) of the pyramidal image. The images depict the proportional decrease of resolution with the increase of the level number in region A. The image have been uploaded with QuPath software.

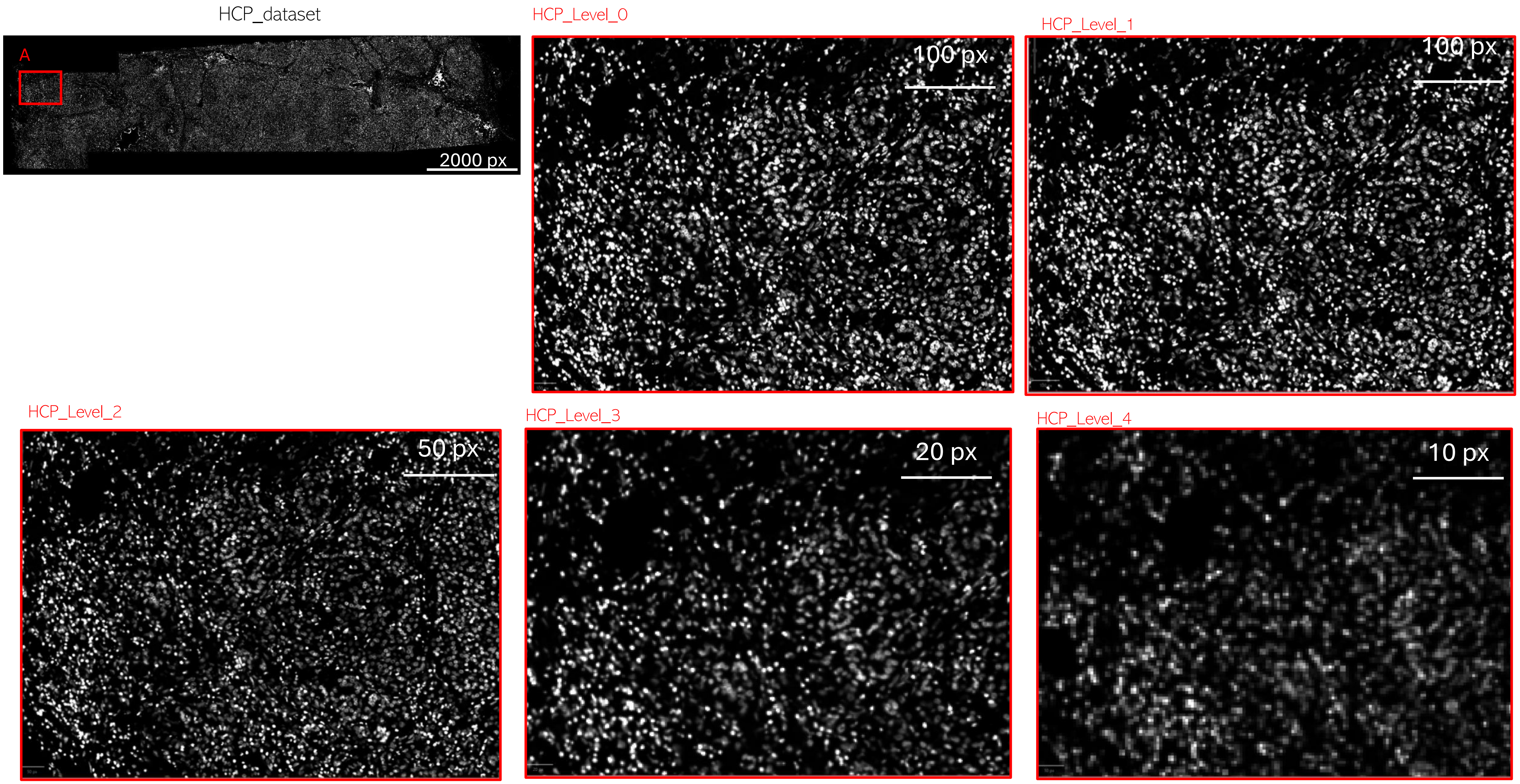

Supplementary Figure 2. Levels resolution in HCP dataset.
dataset images derived from four different level (0,1,2,3,4) of the pyramidal image. The images depict the proportional decrease of resolution with the increase of the level number in region A. The image have been uploaded with QuPath software.

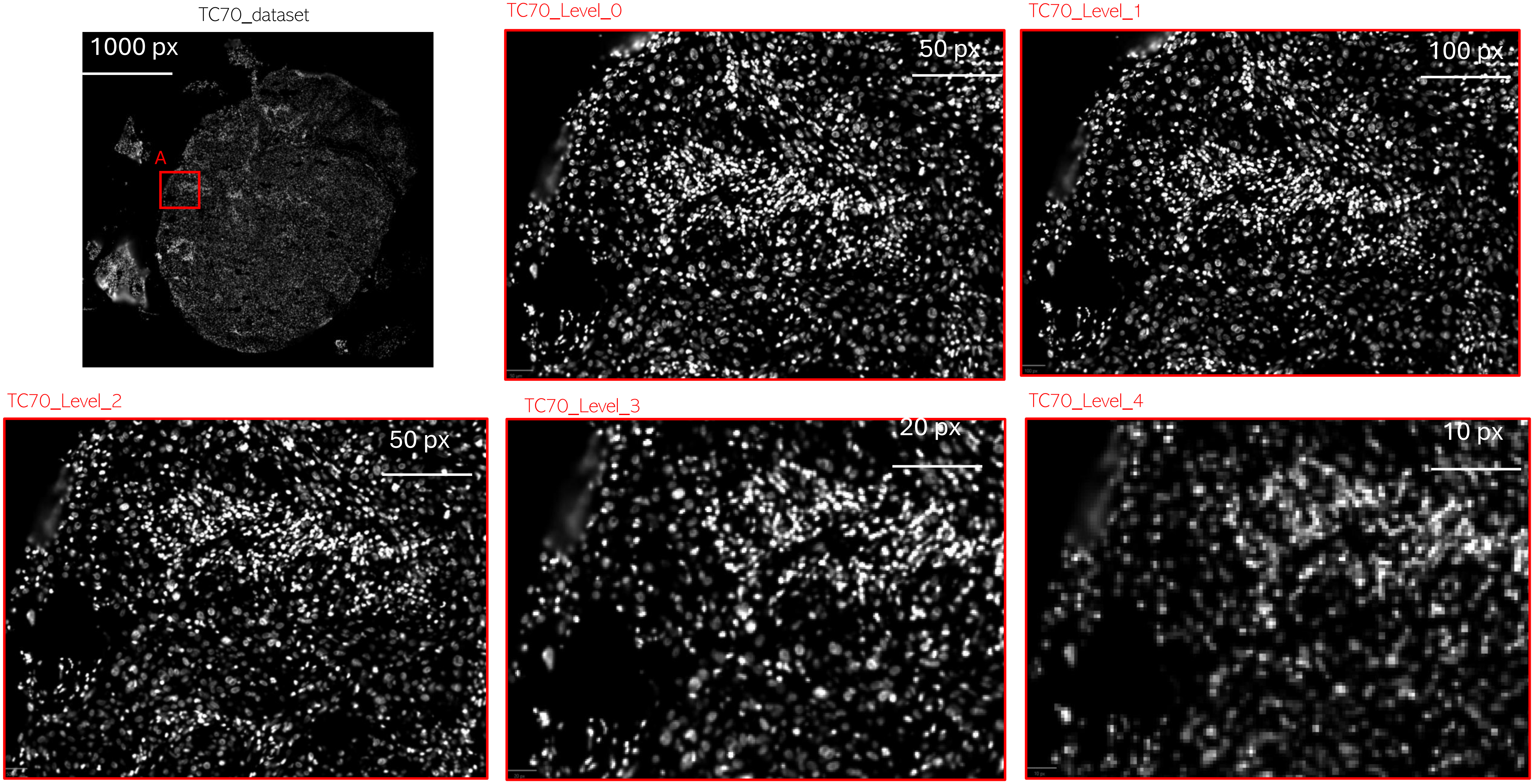

Supplementary Figure 3. Levels resolution in TC70 dataset.
TC70 dataset images derived from four different level (0,1,2,3,4) of the pyramidal image. The images depict the proportional decrease of resolution with the increase of the level number in region A. The image have been uploaded with QuPath software.

Supplementary Figure 4. Quality control metrics in the three datasets.
Distributions of negative control probes (ControlProbe_), negative control codewords (ControlCodeword_), and blank/unassigned codewords (Blank Codeword_) are shown for each dataset (KCP, HCP and TC70) comparing 10x derived data ("CTRL") and STAT3D data. Control counts are displayed on a log10 scale.

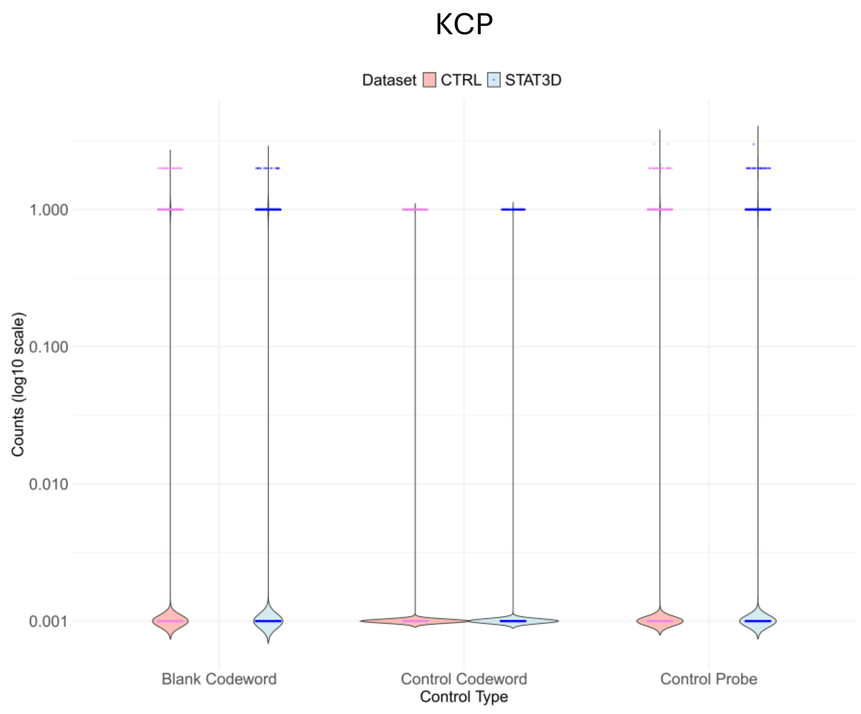

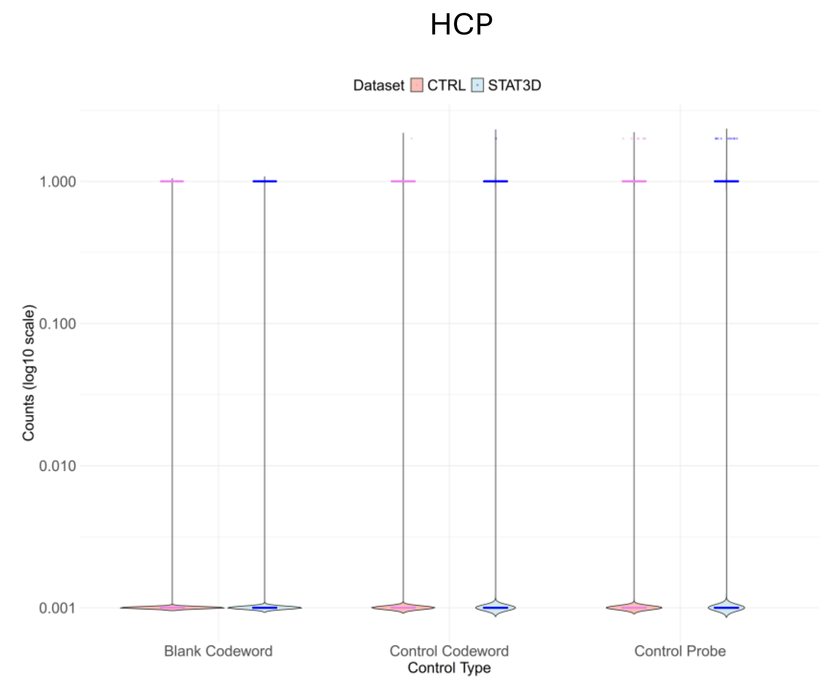

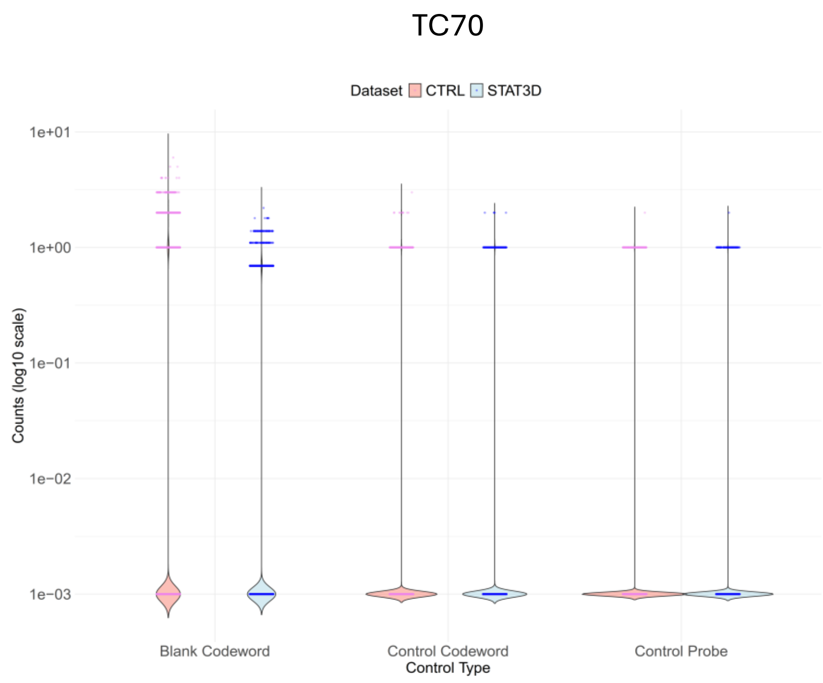

Min. Min.	Q1.1st Qu.	Median. Median	Mean. Mean	Q3.3rd Qu.	Max. Max.	Metric	Dataset
0	0	0	0.017	0	3	Control Probe	STAT3D_KCP
0	0	0	0.0026	0	1	Control Codeword	STAT3D_KCP
0	0	0	0.026	0	2	Blank Codeword	STAT3D_KCP
0	0	0	0.013	0	3	Control Probe	CTRL_KCP
0	0	0	0.0022	0	1	Control Codeword	CTRL_KCP
0	0	0	0.022	0	2	Blank Codeword	CTRL_KCP

Min. Min.	Q1.1st Qu.	Median. Median	Mean. Mean	Q3.3rd Qu.	Max. Max.	Metric	Dataset
0	0	0	0.0068	0	2	Control Probe	STAT3D_HCP
0	0	0	0.0055	0	2	Control Codeword	STAT3D_HCP
0	0	0	0.0015	0	1	Blank Codeword	STAT3D_HCP
0	0	0	0.0033	0	2	Control Probe	CTRL_HCP
0	0	0	0.0026	0	2	Control Codeword	CTRL_HCP
0	0	0	0.0007	0	1	Blank Codeword	CTRL_HCP

Min. Min.	Q1.1st Qu.	Median. Median	Mean. Mean	Q3.3rd Qu.	Max. Max.	Metric	Dataset
0	0	0	0.0034	0	2	Control Probe	STAT3D_TC70
0	0	0	0.0072	0	2	Control Codeword	STAT3D_TC70
0	0	0	0.029	0	2.19	Blank Codeword	STAT3D_TC70
0	0	0	0.0030	0	2	Control Probe	CTRL_TC70
0	0	0	0.0069	0	3	Control Codeword	CTRL_TC70
0	0	0	0.065	0	6	Blank Codeword	CTRL_TC70

Supplementary Figure 5. Feature plot visualization of $LAMP3^{+}$ DCs and cDC1s in the KCP and TC70 datasets.

A. Feature plots displaying the expression levels of CD83, CSF2RA, CCR7 and LAMP3 in KCP dataset across four different analysis settings (CTRL KCP, BAYSOR KCP, 3D 2 Z-STACK STAT3D and SOPA).

B. Feature plots displaying the expression levels of CD83, CSF2RA, CCR7 and LAMP3 in TC70 dataset across four different analysis settings (CTRL TC70, 2D 2 Z-STACK STAT3D, 3D 2 Z-STACK STAT3D and SOPA).

C. Feature plots displaying the expression levels of ITGAX, XCR1, CLEC9A in TC70 dataset across four different analysis settings (CTRL TC70, 2D TC70 2 Z-STACK STAT3D, 3D 2 Z-STACK STAT3D and SOPA).

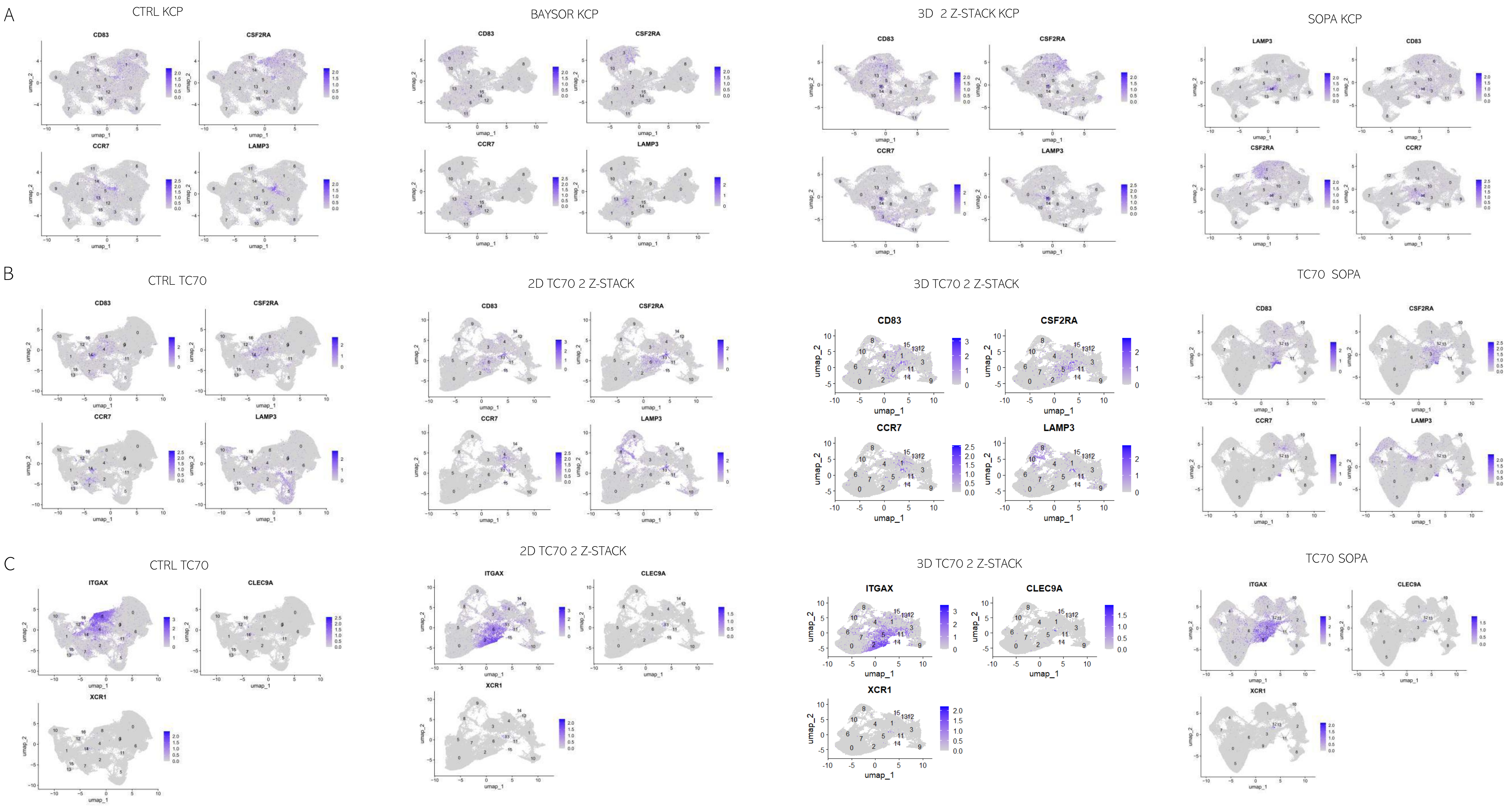

Supplementary Figure 6. Expression patterns of upregulated genes in the TC70 dataset.

Heatmaps displaying the ten top regulated genes with average log2 fold change (avg_log2FC) > 1 in A) TC70 (2D 2 Z-STACK), B) TC70 3D 2 Z-STACK, C) TC70 CTRL and D) TC70 Sopa.

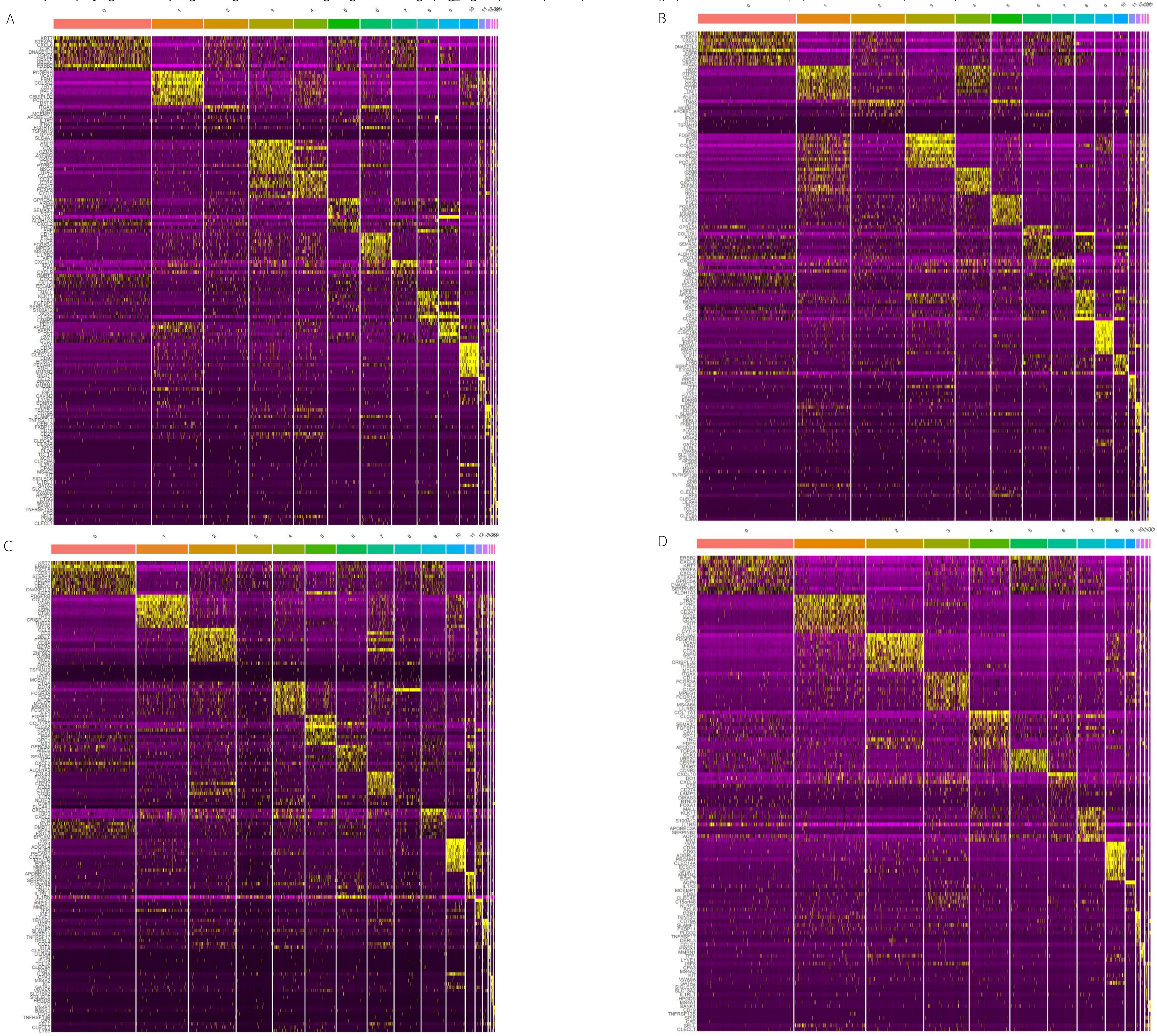

Supplementary Figure 7. Visualization of SingleR-derived cell type prediction scores using the HumanPrimaryCellAtlas and NovershternHematopoieticData reference in the three datasets.
A) Heat maps displaying the prediction scored generated by singleR automated annotation with the “HumanPrimaryCellAtlas” reference in “2 Z-STACK 3D”TC70 dataset. B) Heat maps displaying the prediction scored generated by singleR automated annotation with the “HumanPrimaryCellAtlas” reference in “ALL Z-STACK 2D” HCP dataset. C)Heat maps displaying the prediction scored generated by singleR automated annotation with the “NovershternHematopoieticData” reference” in “1 Z-STACK 2D” KCP dataset.

A

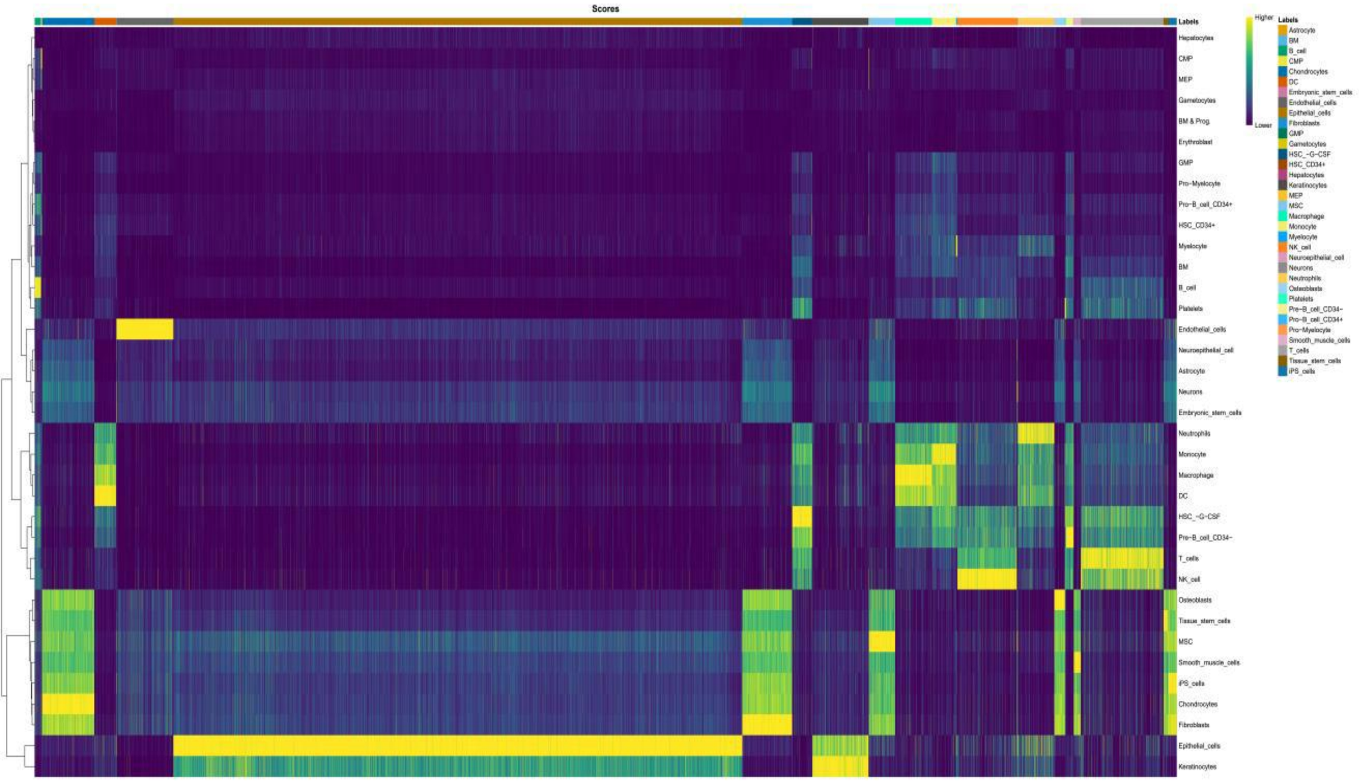

B

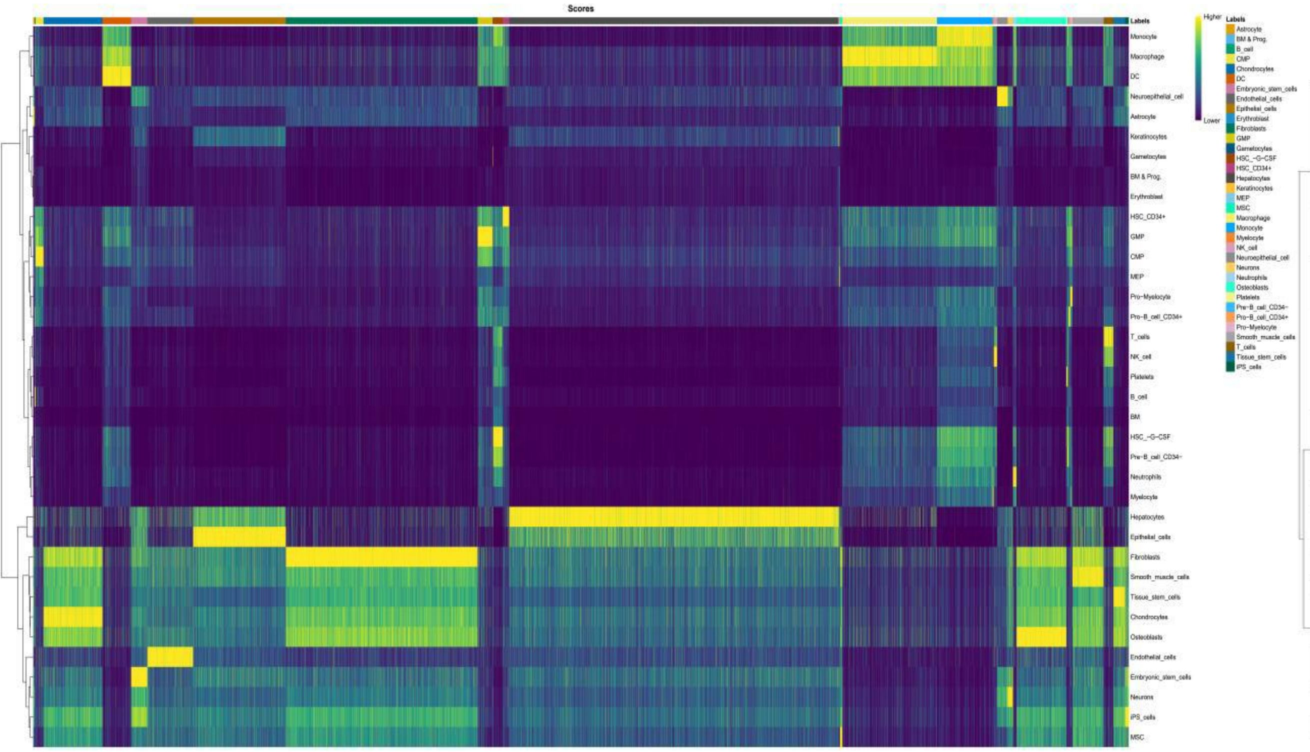

C

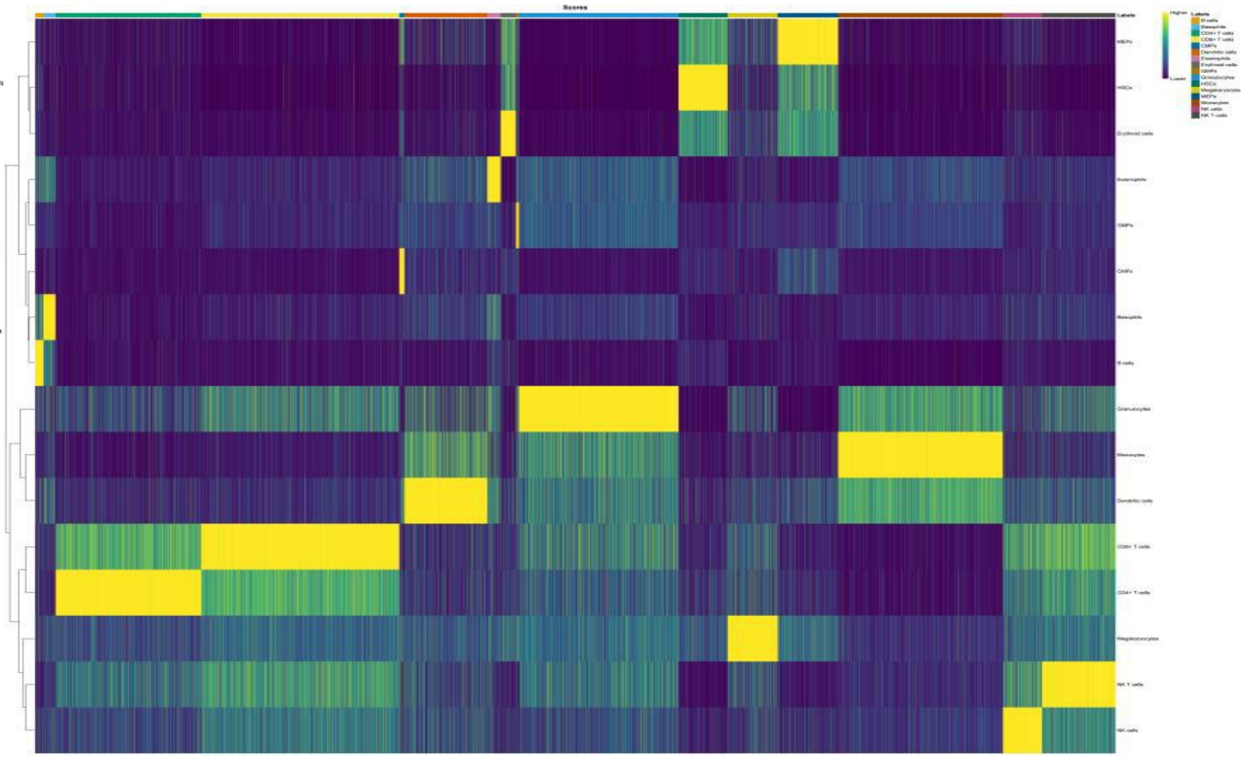

Supplementary Figure 8. Z-stack resolution across level 0 of morphology.ome.tiff images from STAT3D export in the KCP dataset.
Images derived from the “export” folder generated by STAT3D during the image analysis in KCP dataset. Every image displays the resolution of each z-stack within level zero of morphology.ome.tiff.
Scale bar set at 800 μm.

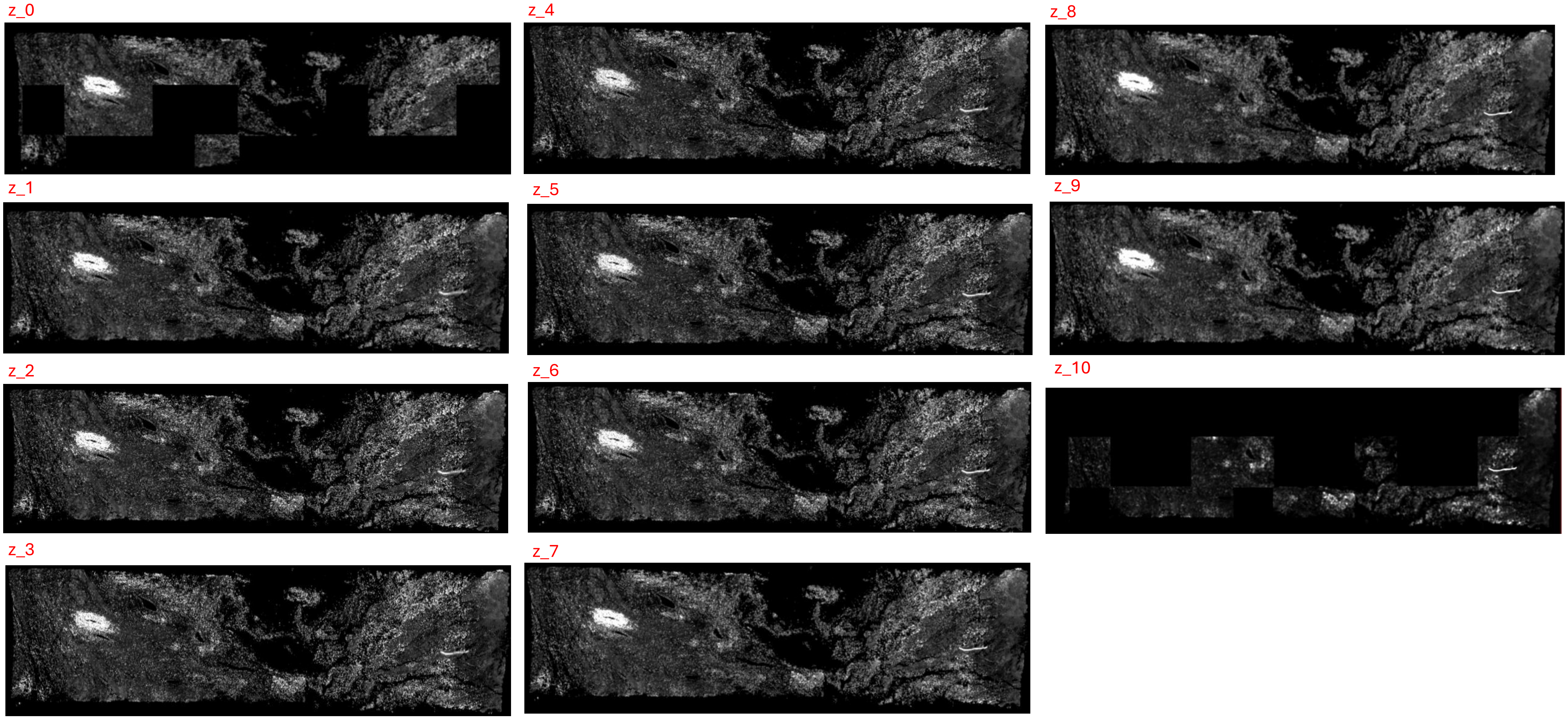

Supplementary Figure 9. Z-stack resolution across level 0 of morphology.ome.tiff images from STAT3D export in the TC70 dataset.
Images derived from the “export” folder generated by STAT3D during the image analysis in TC70 dataset. Every image displays the resolution of each z-stack within level zero of morphology.ome.tiff.
Scale bar set at 400 µm.

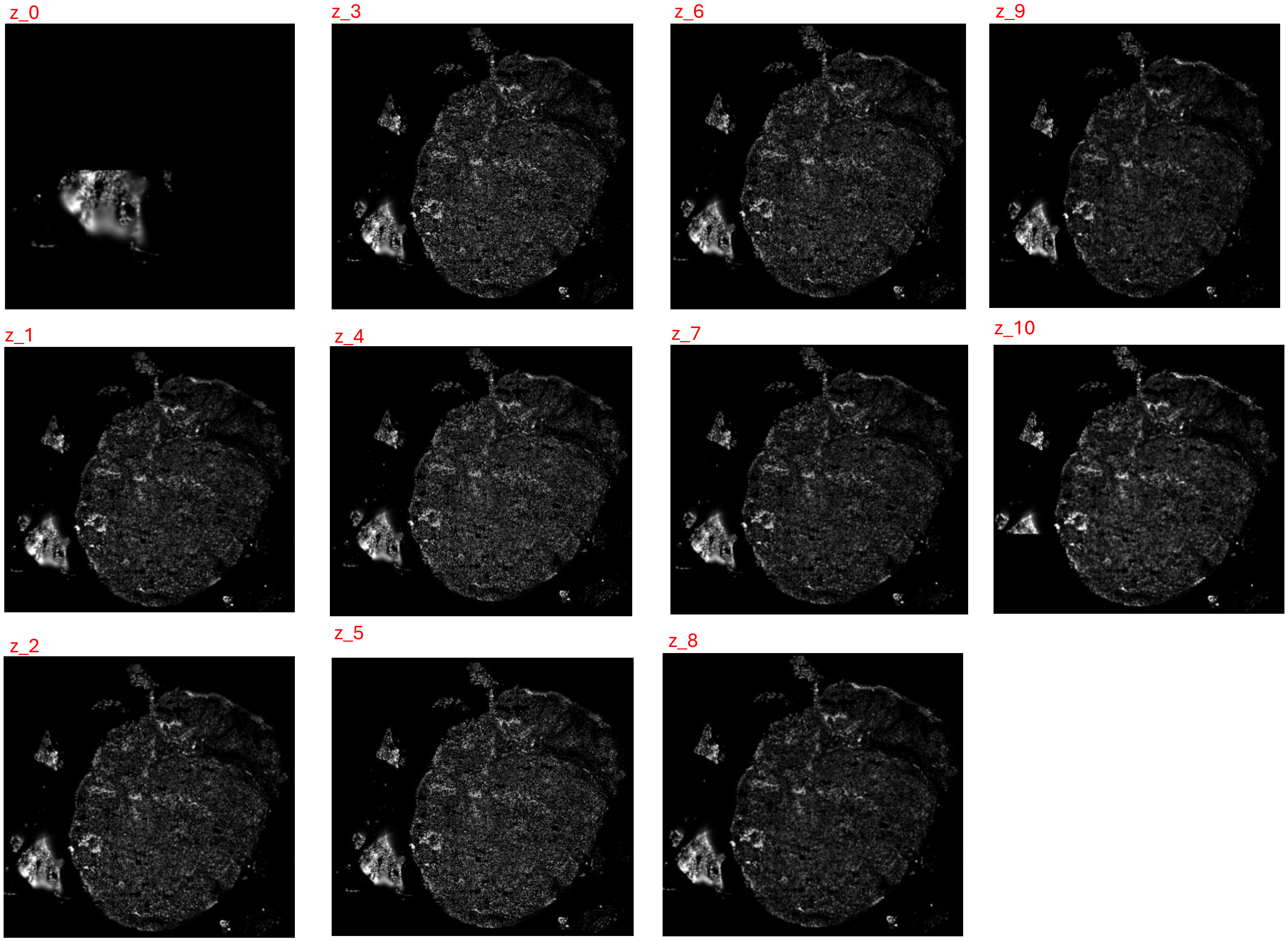

Supplementary Figure 10. Z-stack resolution across level 0 of morphology.ome.tiff images from STAT3D export in the HCP dataset.
Images derived from the "export" folder generated by STAT3D during the image analysis in HCP dataset. Every image displays the resolution of each z-stack within level zero of morphology.ome.tiff.
Scale bar set at 100 μm.

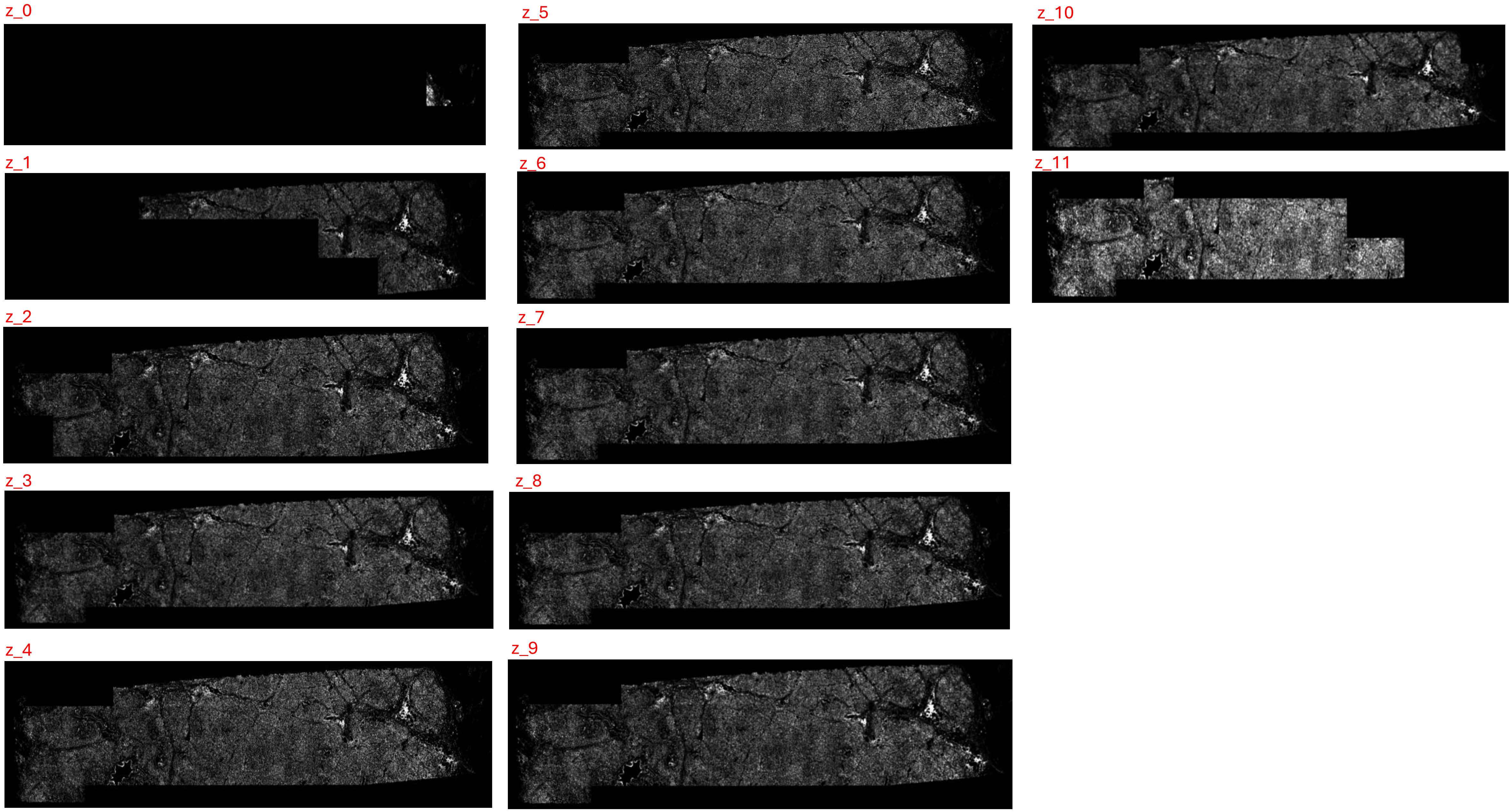

Supplementary Figure 11. Comparison of low-intensity region detection and segmentation in the KCP dataset using Xenium Explorer and STAT3D-QuPath
Image analysis in KCP datasetdataset displaying low intensities regions identified with Xenium Explorer software in CTRL setting (A,C) on morphology.ome.tif image and QuPath Image Software with STAT3D tailored parameters (B,D) in morphology.ome_STAT3D.tif. Orange arrows mark low intensity regions detected as cells. Blue arrows identify cells oversegmented.

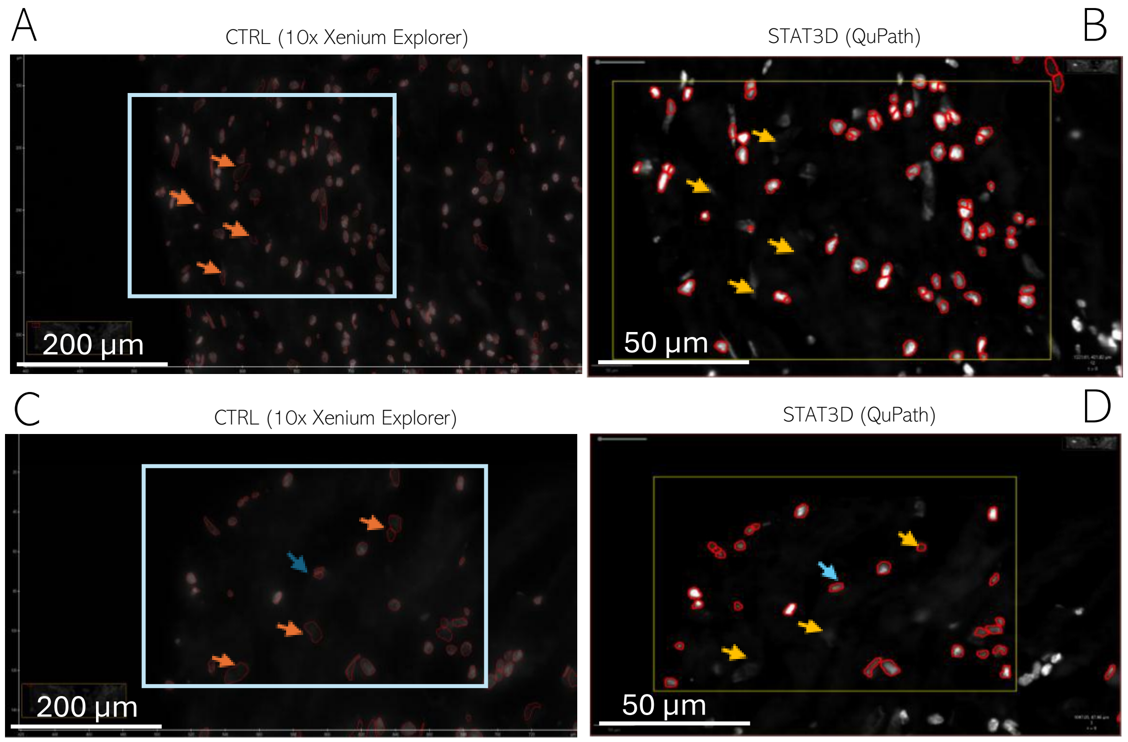

Supplementary Figure 12. Pipelines efficiency for discriminating crowded cell types.
FNN-derived T cells frequencies within 20 µm radii from every B cell in HCP, KCP and TC70 datasets with STAT3D, Sopa and 10x pipelines.

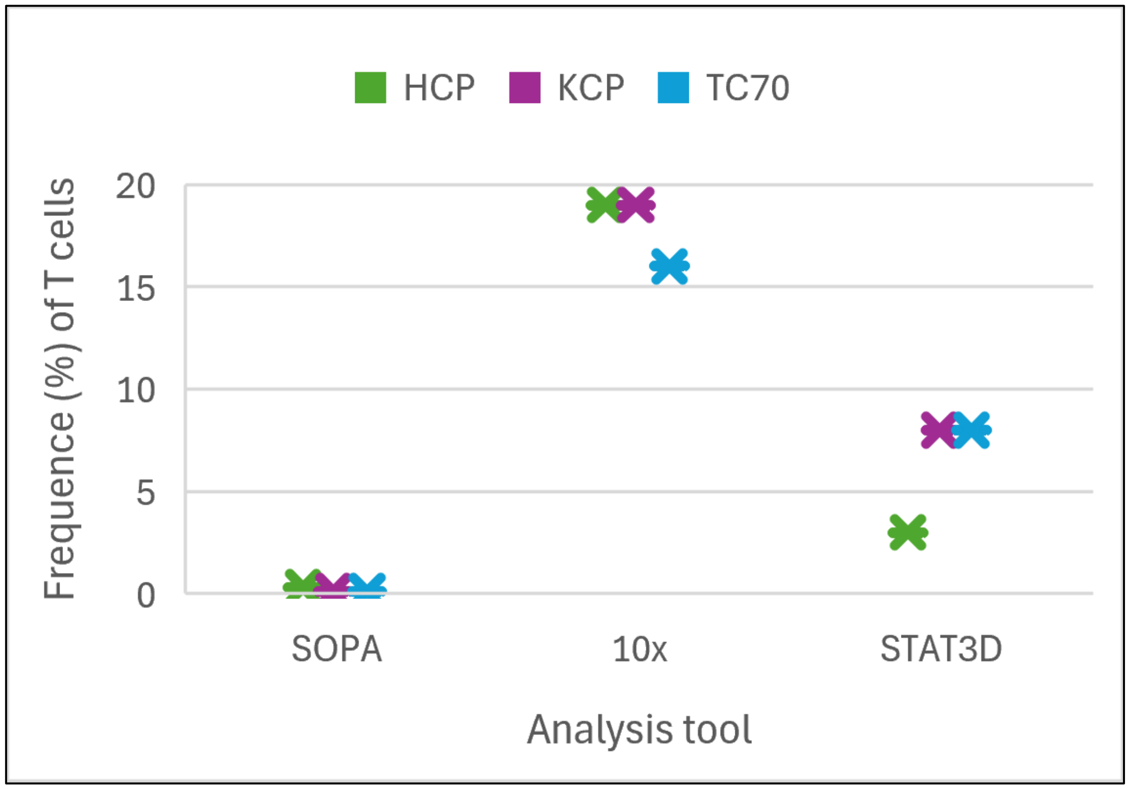

Supplementary Figure 13. Comparison of STAT3D analysis efficacy in HD dataset using different acquisition strategies and analysis pipelines. **(a)** Dot plot displaying average expression levels and percentage of cells expressing macrophages gene signature (MRC1/CD163/CD68/CXCL10) (blue) in HD dataset analysed with seven different computational strategies. **(b)** Dot plot displaying average expression levels and percentage of singleR derived DCs markers (HLA-DQB2, LAMP3, CCR7, CSF2RA) and macrophages marker (CD68) in HD dataset analysed with partially insensitive STAT3D pipeline setup (Random nuclear expansion RNE, Random Z-slice selection RZ). **(c-d)** Spatial UMAP representationdistribution of HD dataset analysed with STAT3D (with MIP setting "HD_ALL_Z_STACK_2D") (c), and RZ setting (z=2) (d). **(e)**Table displaying the diameter measurements with STAT3D and Sopa. **(f)** Table displaying STAT3D parameters in the logic-insensitive settings.

a.

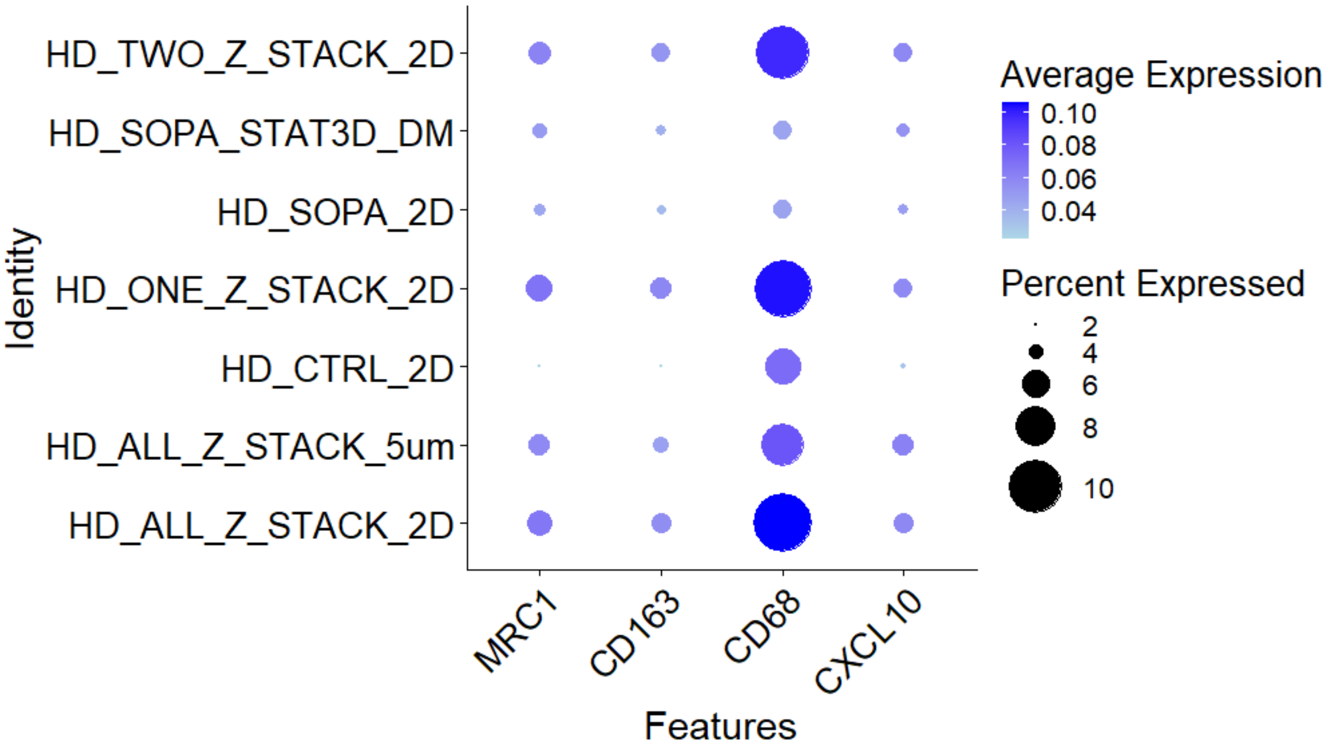

e.

Pipeline	Diameter (px)
STAT3D	27.2
SOPA	30

b.

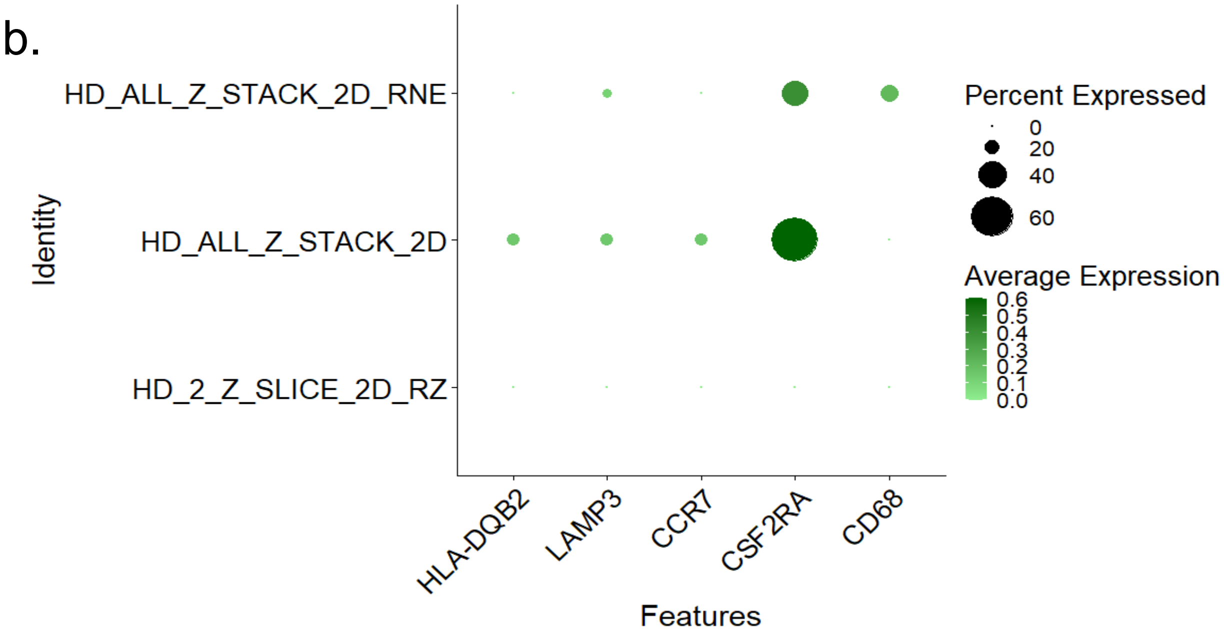

f.

Pipeline setting	Nuclear expansion (µm)	Z-stack
STAT3D full logic	7.79	MIP
STAT3D RNE	15	MIP
STAT3D RZ	7.79	Only z-slice 2

c.

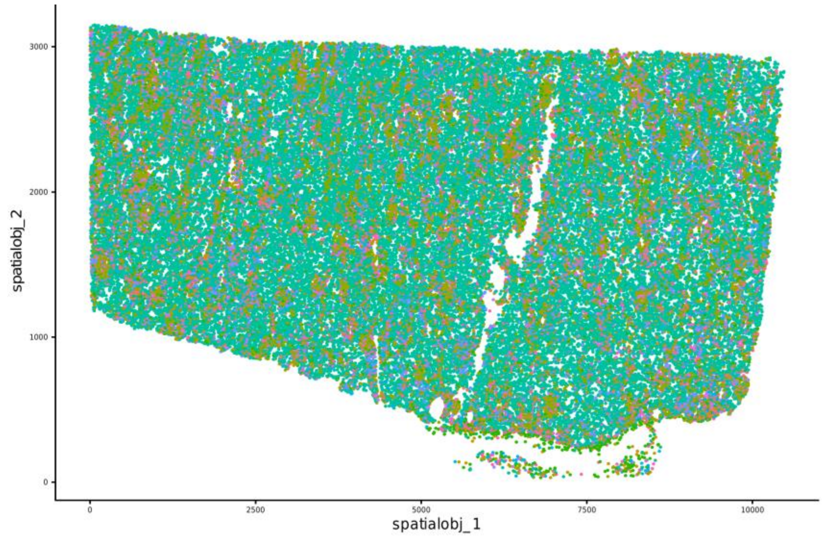

d.

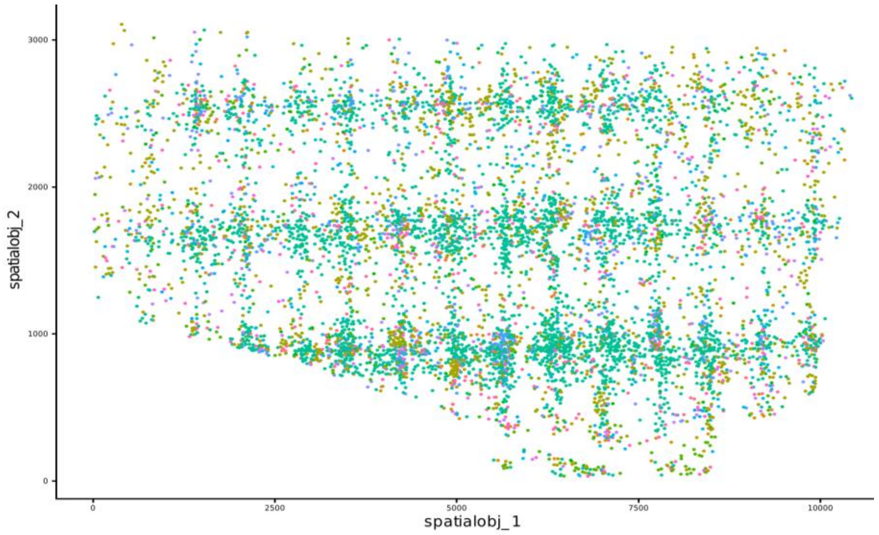

Supplementary Table 1.
Schematic representation of the “z_slice_measurements.csv” STAT3D output file in TC70. The “Z_index” column identifies the total number of z-slices in every image level. The data analysis has been generated with QuPath software.

Image	Object ID	Object type	Name	Classification	Parent	ROI	Centroid X Âµm	Centroid Y Âµm	Z index	Area Âµm^2	Perimeter Âµm
morphology_TC070.ome.tif - Image0	22f54ff9-a4f0-4c72-ac23-aba16b66f4d1	Annotation			Root object (Image)	Rectangle	2431.8	2188.5	0	21288779.7	18481.5
morphology_TC070.ome.tif - Image0	bd89fb01-0391-4d50-9680-a247fe1336cb	Annotation			Root object (Image)	Rectangle	2431.8	2188.5	1	21288779.7	18481.5
morphology_TC070.ome.tif - Image0	214fa143-8363-457c-a63c-a45ec89738fb	Annotation			Root object (Image)	Rectangle	2431.8	2188.5	2	21288779.7	18481.5
morphology_TC070.ome.tif - Image0	ee57d8e7-f1d6-4e43-b597-e171e3bbfe95	Annotation			Root object (Image)	Rectangle	2431.8	2188.5	3	21288779.7	18481.5
morphology_TC070.ome.tif - Image0	3158b4f1-aad1-4bbf-8563-8d8c5d100901	Annotation			Root object (Image)	Rectangle	2431.8	2188.5	4	21288779.7	18481.5
morphology_TC070.ome.tif - Image0	76749b27-630f-4bda-acd4-37f5b38987f6	Annotation			Root object (Image)	Rectangle	2431.8	2188.5	5	21288779.7	18481.5
morphology_TC070.ome.tif - Image0	d5b7c73b-8e3e-4210-a0f9-d3d352cb1de3	Annotation			Root object (Image)	Rectangle	2431.8	2188.5	6	21288779.7	18481.5
morphology_TC070.ome.tif - Image0	cebd4129-e023-4383-b1ab-c4dcb5747f3f	Annotation			Root object (Image)	Rectangle	2431.8	2188.5	7	21288779.7	18481.5
morphology_TC070.ome.tif - Image0	63286651-d5df-4b01-a6fd-2a7632501f9b	Annotation			Root object (Image)	Rectangle	2431.8	2188.5	8	21288779.7	18481.5
morphology_TC070.ome.tif - Image0	d6886395-08df-4d44-95b0-3ef94836466d	Annotation			Root object (Image)	Rectangle	2431.8	2188.5	9	21288779.7	18481.5
morphology_TC070.ome.tif - Image0	ad091fd8-2c15-443e-9f97-90950b0e5a13	Annotation			Root object (Image)	Rectangle	2431.8	2188.5	10	21288779.7	18481.5

Supplementary Table 2.
Schematic representation of the “z_slice_measurements.csv” STAT3D output file in KCP. The “Z_index” column identifies the total number of z-slices in every image level. The data analysis has been generated with QuPath software.

Image	Object ID	Object type	Name	Classification	Parent	ROI	Centroid X Âµm	Centroid Y Âµm	Z index	Area Âµm^2	Perimeter Âµm
morphology_K.ome.tif - Image0	db265c3b-cded-4d6c-a5c9-216c2c56572d	Annotation			Root object (Image)	Rectangle	3925.2	1099.3	0	17259273.2	20097.8
morphology_K.ome.tif - Image0	7777a828-0947-4c2a-995c-5462c4bad2f0	Annotation			Root object (Image)	Rectangle	3925.2	1099.3	1	17259273.2	20097.8
morphology_K.ome.tif - Image0	53f2ec89-5d94-4de1-bdfa-f341bb8f027d	Annotation			Root object (Image)	Rectangle	3925.2	1099.3	2	17259273.2	20097.8
morphology_K.ome.tif - Image0	e8e63baa-2ced-466a-9f8f-c030266060de	Annotation			Root object (Image)	Rectangle	3925.2	1099.3	3	17259273.2	20097.8
morphology_K.ome.tif - Image0	39fd578c-758e-48e0-a9e5-7043fc1b7fb9	Annotation			Root object (Image)	Rectangle	3925.2	1099.3	4	17259273.2	20097.8
morphology_K.ome.tif - Image0	ec9fc7ca-8ded-4f09-9bc3-1a6b187240b3	Annotation			Root object (Image)	Rectangle	3925.2	1099.3	5	17259273.2	20097.8
morphology_K.ome.tif - Image0	5bcebe71-20c0-48fc-9638-13e1e71cb8f5	Annotation			Root object (Image)	Rectangle	3925.2	1099.3	6	17259273.2	20097.8
morphology_K.ome.tif - Image0	e21b3a46-382e-46fd-b5b2-e72ab9fc437f	Annotation			Root object (Image)	Rectangle	3925.2	1099.3	7	17259273.2	20097.8
morphology_K.ome.tif - Image0	8fa97787-9a3e-4af0-8109-f6e857be7caa	Annotation			Root object (Image)	Rectangle	3925.2	1099.3	8	17259273.2	20097.8
morphology_K.ome.tif - Image0	3466e6a2-3e93-4174-811d-825f34ae4248	Annotation			Root object (Image)	Rectangle	3925.2	1099.3	9	17259273.2	20097.8
morphology_K.ome.tif - Image0	f75e7934-76c5-41ad-914e-2fd809c6dec4	Annotation			Root object (Image)	Rectangle	3925.2	1099.3	10	17259273.2	20097.8

Supplementary Table 3.
Schematic representation of the “z_slice_measurements.csv” STAT3D output in HCP. The “Z_index” column identifies the total number of z-slices in every image level. The data analysis has been generated with QuPath software.

Image	Object ID	Object type	Name	Classification	Parent	ROI	Centroid X Âµm	Centroid Y Âµm	Z index	Area Âµm^2	Perimeter Âµm
morphology_Pancreas.ome.tif - Image0	54ed6a4d-9c8a-4a74-9796-dbe1f1fd632e	Annotation			Root object (Image)	Rectangle	5129.1	1461.1	0	29977610.9	26361
morphology_Pancreas.ome.tif - Image0	2ea9fcf8-d02d-41be-83fb-61565a9e64a0	Annotation			Root object (Image)	Rectangle	5129.1	1461.1	1	29977610.9	26361
morphology_Pancreas.ome.tif - Image0	d2906647-20fd-4b8b-b839-fafb35a1ac2d	Annotation			Root object (Image)	Rectangle	5129.1	1461.1	2	29977610.9	26361
morphology_Pancreas.ome.tif - Image0	fdcd4f1f-1574-4955-8976-73d1ae0a4d63	Annotation			Root object (Image)	Rectangle	5129.1	1461.1	3	29977610.9	26361
morphology_Pancreas.ome.tif - Image0	fca26fb7-279d-4487-aa7d-3f341ba0c817	Annotation			Root object (Image)	Rectangle	5129.1	1461.1	4	29977610.9	26361
morphology_Pancreas.ome.tif - Image0	409fc7a9-4933-4537-b7b1-571735dac710	Annotation			Root object (Image)	Rectangle	5129.1	1461.1	5	29977610.9	26361
morphology_Pancreas.ome.tif - Image0	6daf25e3-02be-4353-ac75-60ccf1fbdeb4	Annotation			Root object (Image)	Rectangle	5129.1	1461.1	6	29977610.9	26361
morphology_Pancreas.ome.tif - Image0	b8624562-31cb-4a26-8921-569edc74e8b6	Annotation			Root object (Image)	Rectangle	5129.1	1461.1	7	29977610.9	26361
morphology_Pancreas.ome.tif - Image0	c73c5f2a-e2da-4d15-9c97-de0e91aa3488	Annotation			Root object (Image)	Rectangle	5129.1	1461.1	8	29977610.9	26361
morphology_Pancreas.ome.tif - Image0	e1b2014c-0243-4e7b-a655-8ae6267e406f	Annotation			Root object (Image)	Rectangle	5129.1	1461.1	9	29977610.9	26361
morphology_Pancreas.ome.tif - Image0	32137a3e-49c0-42f0-b425-b33d213bb575	Annotation			Root object (Image)	Rectangle	5129.1	1461.1	10	29977610.9	26361
morphology_Pancreas.ome.tif - Image0	96d83ba8-a7fe-42c9-8db1-fdd4fe802e54	Annotation			Root object (Image)	Rectangle	5129.1	1461.1	11	29977610.9	26361
morphology_Pancreas.ome.tif - Image0	3d2e83ed-d21f-42c3-b234-71e7afb8a438	Annotation			Root object (Image)	Rectangle	5129.1	1461.1	12	29977610.9	26361

Supplementary Table 4.
Classification of multiplex immunofluorescence Opal marker combinations and corresponding cellular or regional annotations.

Single Opal	Annotation
Opal 480	IDO1+ cells
Opal 520	LAMP3+ cells
Opal 570	CD8+ cells
Opal 620	PanCk+ cells
Opal 690	CCR7+ cells

Two Opals Combination	Annotation
Opal 480: Opal 520	IDO1/LAMP3 DCs
Opal 480: Opal 570	IDO1/CD8 region
Opal 570: Opal 520	CD8/LAMP3 region
Opal 620: Opal 480	PanCk/IDO1
Opal 620: Opal 570	PanCk/CD8 region
Opal 620: Opal 690	PanCk/CCR7 regions
Opal 690: Opal 480	CCR7/IDO1 DCs
Opal 690: Opal 520	CCR7/LAMP3 DCs
Opal 690: Opal 570	CCR7/CD8 region

Three Opals Combination	Annotation
Opal 480: Opal 570: Opal 520	IDO1/LAMP3 DCs and CD8+ cells region
Opal 620: Opal 480: Opal 570	PanCk/IDO1/CD8 region
Opal 620: Opal 570: Opal 520	PanCk/CD8/LAMP3 region
Opal 620: Opal 690: Opal 480	PanCk+ cells and CCR7/IDO1 DCs region
Opal 620: Opal 690: Opal 570	PanCk/CCR7/CD8 region
Opal 690: Opal 480: Opal 520	CCR7/IDO1/LAMP3 DCs
Opal 690: Opal 480: Opal 570	CCR7/IDO1 DCs and CD8+ cells region
Opal 690: Opal 570: Opal 520	CCR7/LAMP3 DC and CD8+ cells region

Laplacian scores of the first 7 z-stack images within the first four levels of the image pyramid in TC70, HCP

Level 0 TC70
Sharpness score for slice 0: 300.1276036902101
Sharpness score for slice 1: 3880.8824612073545
Sharpness score for slice 2: 3899.652894463895
Sharpness score for slice 3: 3951.3948229102016
Sharpness score for slice 4: 4828.587284810162
Sharpness score for slice 5: 4919.272185175579
Sharpness score for slice 6: 4026.844142556266
Sharpness score for slice 7: 3959.827548341905

Level 1 TC70
Sharpness score for slice 0: 18.694831052746235
Sharpness score for slice 1: 182.53989691109035
Sharpness score for slice 2: 202.6471310507203
Sharpness score for slice 3: 415.26433515889465
Sharpness score for slice 4: 4720.774076206709
Sharpness score for slice 5: 4681.219487091283
Sharpness score for slice 6: 637.5835582324116
Sharpness score for slice 7: 343.1763016410513

Level 2 TC70
Sharpness score for slice 0: 12.193159471640964
Sharpness score for slice 1: 107.69734467769675
Sharpness score for slice 2: 320.6301028022802
Sharpness score for slice 3: 2431.6161062334672
Sharpness score for slice 4: 20641.640568485163
Sharpness score for slice 5: 19405.728880275223
Sharpness score for slice 6: 3091.1406583619337
Sharpness score for slice 7: 946.9322849807393

Level 3 TC70
Sharpness score for slice 0: 28.37600848970771
Sharpness score for slice 1: 696.5596950144214
Sharpness score for slice 2: 2810.394011754516
Sharpness score for slice 3: 18794.92334563736
Sharpness score for slice 4: 76311.42192471343
Sharpness score for slice 5: 70881.70070816462
Sharpness score for slice 6: 19270.552615239296
Sharpness score for slice 7: 5245.2934292603295

Level 4 TC70
Sharpness score for slice 0: 101.60786017483075
Sharpness score for slice 1: 5039.05731011933
Sharpness score for slice 2: 19026.657223774644
Sharpness score for slice 3: 71841.97214762247
Sharpness score for slice 4: 155692.8923469498
Sharpness score for slice 5: 147759.6494293405
Sharpness score for slice 6: 67350.12035971766
Sharpness score for slice 7: 22767.000987913496

Level 0 HCP
Sharpness score for slice 0: 51.79263716356576
Sharpness score for slice 1: 2387.574529015618
Sharpness score for slice 2: 5448.643514066277
Sharpness score for slice 3: 5800.21962301834
Sharpness score for slice 4: 6348.834476712019
Sharpness score for slice 5: 9181.052210608499
Sharpness score for slice 6: 9545.358663121953
Sharpness score for slice 7: 6572.07735384856

Level 1 HCP
Sharpness score for slice 0: 4.067403547661312
Sharpness score for slice 1: 124.20450443793275
Sharpness score for slice 2: 270.8284309560924
Sharpness score for slice 3: 359.7007963775137
Sharpness score for slice 4: 2269.310182653542
Sharpness score for slice 5: 10691.010542869464
Sharpness score for slice 6: 11084.167681862476
Sharpness score for slice 7: 2575.891660600967

Level 2 HCP
Sharpness score for slice 0: 3.548234620840639
Sharpness score for slice 1: 70.57987127624052
Sharpness score for slice 2: 191.65762441660954
Sharpness score for slice 3: 829.1823571073804
Sharpness score for slice 4: 8585.870903810765
Sharpness score for slice 5: 32266.12896119198
Sharpness score for slice 6: 31800.018893325574
Sharpness score for slice 7: 8527.25811775727

Level 3 HCP
Sharpness score for slice 0: 7.74481004645177
Sharpness score for slice 1: 249.48525353843135
Sharpness score for slice 2: 1075.848263356299
Sharpness score for slice 3: 6042.330045032198
Sharpness score for slice 4: 36398.31803593653
Sharpness score for slice 5: 95612.37914612111
Sharpness score for slice 6: 90984.72094950457
Sharpness score for slice 7: 32951.86159067823

Level 4 HCP
Sharpness score for slice 0: 25.453144455435265
Sharpness score for slice 1: 1498.411066214352
Sharpness score for slice 2: 7349.060910385549
Sharpness score for slice 3: 29286.193850086973
Sharpness score for slice 4: 94913.44768898927
Sharpness score for slice 5: 177212.53415905833
Sharpness score for slice 6: 170529.07644100962
Sharpness score for slice 7: 86725.34254739125

Level 0 KCP
Sharpness score for slice 0: 2081.8790790389157
Sharpness score for slice 1: 3636.55595357666
Sharpness score for slice 2: 3644.6796983246113
Sharpness score for slice 3: 3761.0238221128125
Sharpness score for slice 4: 4566.338238176634
Sharpness score for slice 5: 3874.014836511885
Sharpness score for slice 6: 3658.785143529599
Sharpness score for slice 7: 3646.9564156440115

Level 1 KCP
Sharpness score for slice 0: 2081.8790790389157
Sharpness score for slice 1: 3636.55595357666
Sharpness score for slice 2: 3644.6796983246113
Sharpness score for slice 3: 3761.0238221128125
Sharpness score for slice 4: 4566.338238176634
Sharpness score for slice 5: 3874.014836511885
Sharpness score for slice 6: 3658.785143529599
Sharpness score for slice 7: 3646.9564156440115

Level 2 KCP
Sharpness score for slice 0: 46.578481021668885
Sharpness score for slice 1: 82.22629861833913
Sharpness score for slice 2: 352.9445198009446
Sharpness score for slice 3: 3856.758970022478
Sharpness score for slice 4: 13349.914372001698
Sharpness score for slice 5: 4372.289956036547
Sharpness score for slice 6: 461.8341424034069
Sharpness score for slice 7: 87.20455298119903

Level 3 HCP
Sharpness score for slice 0: 139.6001874632799
Sharpness score for slice 1: 526.1553485152198
Sharpness score for slice 2: 3096.250901632409
Sharpness score for slice 3: 20176.905308747067
Sharpness score for slice 4: 43059.97730116665
Sharpness score for slice 5: 20467.45317943693
Sharpness score for slice 6: 3424.54595168254
Sharpness score for slice 7: 553.4819704224203

Level 4 HCP
Sharpness score for slice 0: 825.2787930018685
Sharpness score for slice 1: 3977.4584361413513
Sharpness score for slice 2: 16099.152927028723
Sharpness score for slice 3: 50123.60986191122
Sharpness score for slice 4: 76422.6913032426
Sharpness score for slice 5: 49780.08646466671
Sharpness score for slice 6: 16251.0730726866
Sharpness score for slice 7: 4130.478945695469
